# Optimized Identification of Advanced Chronic Kidney Disease and Absence of Kidney Disease by Combining Different Electronic Health Data Resources and by Applying Machine Learning Strategies

**DOI:** 10.3390/jcm9092955

**Published:** 2020-09-12

**Authors:** Christoph Weber, Lena Röschke, Luise Modersohn, Christina Lohr, Tobias Kolditz, Udo Hahn, Danny Ammon, Boris Betz, Michael Kiehntopf

**Affiliations:** 1 Department of Clinical Chemistry and Laboratory Diagnostics and Integrated Biobank Jena (IBBJ), Jena University Hospital, 07747 Jena, Germany; christoph.weber@med.uni-jena.de (C.W.); lena.marie.roeschke@uni-jena.de (L.R.); 2 Jena University Language & Information Engineering (JULIE) Lab, Friedrich Schiller University Jena, 07743 Jena, Germany; luise.modersohn@uni-jena.de (L.M.); christina.lohr@uni-jena.de (C.L.); tbs.kldtz@gmail.com (T.K.); udo.hahn@uni-jena.de (U.H.); 3 Data Integration Center, Jena University Hospital, 07743 Jena, Germany; danny.ammon@med.uni-jena.de

**Keywords:** chronic kidney disease (CKD), no known kidney disease (NKD), ICD-10 billing codes, phenotyping, electronic health record (EHR), estimated glomerular filtration rate (eGFR), machine learning (ML), generalized linear model network (GLMnet), random forest (RF), artificial neural network (ANN), clinical natural language processing (clinical NLP), discharge summaries, laboratory values, area under the receiver operating characteristic (AUROC), area under the precision-recall curve (AUCPR)

## Abstract

Automated identification of advanced chronic kidney disease (CKD ≥ III) and of no known kidney disease (NKD) can support both clinicians and researchers. We hypothesized that identification of CKD and NKD can be improved, by combining information from different electronic health record (EHR) resources, comprising laboratory values, discharge summaries and ICD-10 billing codes, compared to using each component alone. We included EHRs from 785 elderly multimorbid patients, hospitalized between 2010 and 2015, that were divided into a training and a test (n = 156) dataset. We used both the area under the receiver operating characteristic (AUROC) and under the precision-recall curve (AUCPR) with a 95% confidence interval for evaluation of different classification models. In the test dataset, the combination of EHR components as a simple classifier identified CKD ≥ III (AUROC 0.96[0.93–0.98]) and NKD (AUROC 0.94[0.91–0.97]) better than laboratory values (AUROC CKD 0.85[0.79–0.90], NKD 0.91[0.87–0.94]), discharge summaries (AUROC CKD 0.87[0.82–0.92], NKD 0.84[0.79–0.89]) or ICD-10 billing codes (AUROC CKD 0.85[0.80–0.91], NKD 0.77[0.72–0.83]) alone. Logistic regression and machine learning models improved recognition of CKD ≥ III compared to the simple classifier if only laboratory values were used (AUROC 0.96[0.92–0.99] vs. 0.86[0.81–0.91], *p* < 0.05) and improved recognition of NKD if information from previous hospital stays was used (AUROC 0.99[0.98–1.00] vs. 0.95[0.92–0.97]], *p* < 0.05). Depending on the availability of data, correct automated identification of CKD ≥ III and NKD from EHRs can be improved by generating classification models based on the combination of different EHR components.

## 1. Introduction

Chronic kidney disease (CKD) is a major public health concern characterized by an increasing prevalence and associated with a high level of morbidity and mortality [1,2]. Correct identification of CKD is crucial, e.g., for appropriate dosing of drugs and for early intervention, including the prevention of progression [3]. For clinical research, accurate identification of CKD or absence of kidney disease (NKD = no known kidney disease) is essential for clinical trials and epidemiological studies. In this context, a particular challenge is to store samples from hospitalized patients with known kidney status in clinical biorepositories, as part of Healthcare-Integrated Biobanking (HIB). At the time point of sample selection and storage, only a limited range of information regarding the respective patient phenotype is available.

Administrative data such as ICD-10 billing codes are often used in research trails to identify patients with CKD [4]. However, administrative databases are not maintained with the primary purpose of supporting research; thus, it might be that, e.g., mild impairment of kidney function will be underrepresented because they cannot be billed [5]. Indeed, many studies have demonstrated that ICD-10 billing codes considerably underestimate the prevalence of CKD [6]. Moreover, there is no ICD-10 billing code for NKD, as the purpose of ICD-10 billing codes is to indicate the presence of a disease.

Electronic health records (EHRs) are a promising source for the diagnosis or exclusion of CKD. EHRs contain structured data (laboratory values, epidemiological data) and unstructured data (narrative discharge summaries).

The laboratory assessment of kidney function is based on an equation to estimate the glomerular filtration rate (GFR) [3]. This equation, Chronic Kidney Disease Epidemiology Collaboration (CKD-EPI), includes the blood creatinine level, age, sex and ethnicity [7]. According to the Kidney Disease: Improving Global Outcomes (KDIGO) definition, CKD Stage III and higher can be diagnosed by an eGFR below 60 mL/min/1.73m^2^ for a time period of at least 90 days [3]. However, previous laboratory data on hospitalized patients are often not fully available, e.g., they were recorded in other hospitals or in outpatient clinics.

Unstructured data such as discharge summaries can fill the gap of missing medical information. Letters are available in a digital form for every hospitalized patient and often contain complementary information, not only about the current hospital stay, but also about the clinical history of the patient including chronic diseases. Information can be extracted from narrative discharge summaries for example by reusing SNOMED CT codes from EHRs [8], screening the letters for disease-specific keywords [9,10], or using ML based natural language processing (NLP) technology for ICD-10 billing codes [11] or SNOMED CT [12] coding, named entity recognition [13], or relation extraction [14].

Data analysis from EHRs can be performed in a rule-based format for example by strictly adhering to the KDIGO definition of CKD ≥ III. In recent years, various machine learning (ML) methods have been applied to improve the automated recognition of chronic kidney disease, using mainly laboratory values and demographic information [15,16,17,18,19,20]. However, to the best of our knowledge, no study specifically targeted advanced CKD ≥ III or NKD.

In this study, we hypothesize that combining structured (laboratory values, ICD-10 billing codes) and unstructured (discharge summaries) information from EHRs and applying ML for data analysis can reliably distinguish between patients with advanced CKD (stage ≥ III) and patients with no known kidney disease (NKD) in different scenarios of data availability.

## 2. Materials and Methods

### 2.1. Study Population

The dataset of this retrospective study has been derived from the Jena Part of the 3000 PA text corpus of the Smart Medical Information Technology for Healthcare (SMITH) consortium (part of the Medical Informatics Initiative founded by the German Federal Ministry of Education and Research) [21,22,23]. The dataset consisted of EHRs from 785 individuals who were from European descent and had an index hospital stay for at least five days on a ward for internal medicine or in an intensive care unit between 2010 and 2015. No individual deceased during the index hospital stay. At the time point of retrospective data collection, all individuals were deceased. The EHRs included discharge summaries, laboratory values and ICD-10 billing codes. The study was approved by the local ethics committee (4639-12/15); data were collected retrospectively and anonymized, individual-level informed consent of participants was waived by the ethics review board. The study was also approved by the data protection officer of Jena University Hospital.

### 2.2. Classification of CKD and NKD by ICD-10 Billing Codes

For classification of CKD and NKD, ICD-10 billing codes of the index hospital stay, extracted from the hospital accounting system and from hospital discharge summaries, were used. For extraction of kidney diseases from discharge summaries the Health Discovery text mining tool v5.7.0 from Averbis (https://health-discovery.io/) was applied using the discharge pipeline with default settings to extract basic medical information (detailed information can be found in the Averbis Health Discovery User Manual Version 5.7, 4 December 2018). Subsequently, a Python script was applied to extract the ICD-10 billing codes from these output files. ICD-10 billing codes for CKD classification were used according to ICD-10 billing codes for moderate to severe kidney disease from the Charlson comorbidity index [24] (Supplementary Materials). For the definition of no kidney disease (NKD), none of these codes as well as further ICD-10 billing codes for kidney disease published by the Centers for Disease Control and Prevention (CDC, http://www.cdc.gov/ckd) (Supplementary Materials) should be present.

### 2.3. Laboratory and Demographic Data

Laboratory values and demographics of the patients were extracted from the laboratory information system (LIS) of the University Hospital of Jena. The following values were considered in the analysis and classification of the study cohort:

- Numerical variables: age, eGFR at admission, eGFR at discharge, eGFR over index hospital stay. Measurements of albumin in urine were available in less than 5% of the cohort and therefore excluded from further analysis.
- Categorical variable: sex.

Descriptive statistics were reported as the mean [SD] or median [I quartile–III quartile] for continuous variables and absolute numbers (percentages) for categorical variables.

### 2.4. Classification of CKD and NKD by Blood Creatinine and eGFR

In order to define CKD and NKD by laboratory values from the current hospital index stay, we created the following rules. If all eGFR values during the index stay were below 60 mL/min/1.73m^2^, the case was assigned to CKD. If all eGFR values during the index hospital stay were above 60 mL/min/1.73m^2^ and there was no presence of AKI (definition see below), the case was assigned to NKD.

### 2.5. Classification of CKD and NKD by Manual Review

CKD stage III or higher was defined according to the KDIGO guidelines. This included an eGFR, based on the formula CKD-EPI [7], which had to be less than 60 mL/min/1.73 m^2^ for at least 3 months (90 days) or by an additional proof of kidney damage [3].

We defined NKD, adapted from James et al. [25], as the complete absence of GFR less than 60 mL/min/1.73m^2^, stable serum creatinine measurements, e.g., no fulfillment of acute kidney disease criteria, median absence of proteinuria when multiple measurements were made before and the absence of AKI in patient laboratory history. AKI was present, if serum creatinine had increased by more than 26.5 mmol/L within 48 h or increased more than 1.5-fold over 7 days [26]. In addition, adapted from the publication by Duff et al. [27], we included AKI recovery defined as a decline in creatinine for more than 33% over 7 days.

All cases were reviewed by an advanced medical student and a physician to assess the underlying kidney status based on individual EHRs, including discharge summaries, ICD-10 billing codes and laboratory test results performed before, subsequent to, and during the index hospital stay. Of note, for clarification of difficult cases, the reviewers used information not available to the rule-based or statistical algorithms (e.g., laboratory values after index hospital stay). The review was used as a reference standard for comparison with automated classification.

### 2.6. Dataset for the Machine Learning Methods

The dataset used for logistic regression and the different ML models is composed of 11 to 19 different categorical and numerical variables. Three of them are derived variables to improve classification.

1. Numerical variables: age; first eGFR of the index hospital stay; last eGFR of the index hospital stay; time difference between the first and last blood measurement of the index hospital stay as an indicator for the length of hospital stay; mean eGFR over index hospital stay; mean eGFR over all available laboratory values.
2. Due to the varying distribution of eGFR measurements, additionally derived numerical variables were defined for usage in ML algorithms: the ratio between the number of hospital visits with eGFR measurements and the number of total visits; the ratio between the number of total eGFR measurements and hospital visits with eGFR measurements; the ratio between the number of eGFR measurements lower than 60 mL/min/1.73 m^2^ and hospital visits with eGFR measurements.
3. Categorical variables: sex; occurrence of AKI and AKI recovery over laboratory history; occurrence of AKI and AKI recovery over index stay.

All of these variables were used in all ML models. Further categorical variables, listed below, were added in different combinations, as described in the results.

CKD: eGFR at admission below 60 mL/min/1.73 m^2^ (eGFR_admission), eGFR at discharge below 60 mL/min/1.73 m^2^ (eGFR_discharge), and all eGFR measurements during index stay below 60 mL/min/1.73 m^2^ (eGFR).

NKD: eGFR at admission above 60 mL/min/1.73 m^2^ (eGFR_admission), eGFR at discharge above 60 mL/min/1.73 m^2^ (eGFR_discharge), eGFR always above 60 mL/min/1.73 m^2^ (eGFR_history), all eGFR during index stay above 60 mL/min/1.73 m^2^ (eGFR); classification by ICD-10 billing codes (ICD); classification by ICD-10 codes from discharge summaries.

### 2.7. Classification of CKD and NKD Using Machine Learning Methods

We applied three different ML methods—generalized linear model via penalized maximum likelihood (GLMnet) [28], random forests (RF) [29] and artificial neural network (ANN) [30]. These are all well-established approaches that represent different types of ML methods.

GLMnet is a statistical method in which different models generalize to the concept of a penalty parameter and in which different models have different loss functions. A penalty parameter constrains the size of the model coefficients such that the only way the coefficients can increase is if a comparable decrease in the models loss function is experienced. A loss function essentially calculates how poorly a model is performing by comparing what the model is predicting with the actual value it is supposed to output. If both values are very similar, the loss value will be very low. There are three common penalty parameters (ridge regression, lasso penalty, elastic-net penalty). We used the elastic-net penalty which is controlled by the *alpha* parameter. It bridges the gap between the ridge regression (alpha = 0), which is good for retaining all features while reducing the noise that less influential variables may create and the lasso (alpha *=* 1) penalty, which actually excludes features from the model.

Like a simple rule-based decision tree, random forests are tree-based models and part of a class of non-parametric algorithms that work by partitioning the feature space into a number of smaller regions. The predictions are obtained by fitting a simpler model in each region. Random forests use the same principles as bagging trees, which grow many trees (*ntree*) on bootstrapped copies of the training data, and extend it with an additional random component through split-variable randomization, where each time a split is to be performed the search for the split variable is limited to a random subset (*mtry*) of the original features.

Artificial neural networks are designed to simulate the biological neural networks of animal brains. They process input examples of a given task and map them against the desired output by forming probability-weighted associations between the two, storing these in the net data structure itself. In its basic form a neural network has three layers. An input layer which consists of all of the original input features, a hidden layer where the majority of the learning process takes place and an output layer [31].

The dataset was randomly split into 80% training and 20% test data. The prevalence for CKD or NKD respectively was similar in the two datasets (Supplementary Materials).

To properly adapt the ML algorithms, we optimized the hyperparameters that are used to control the learning process of a model and cannot be directly estimated from the data. We used a grid search method, which is simply an exhaustive search through a manually specified subset of the hyperparameter space of the learning algorithm. We specified these hyperparameters for every type of model, trailed all combinations and selected the model with the best results (see Supplementary Materials for details). For the GLMnet, the regularization parameter *lambda*, which controls the overall strength of the penalty term and helps to control the model from overfitting to the training data, was calculated during a pre-training of the model. Subsequently the best alpha parameter was determined. It ranges between [0,1] and was divided into steps of 0.1.

Random forest was tuned on the *mtry* parameter in a range between [1,18] depending on the number of features of the model, divided into steps of 1. The *ntree* parameter was set to its default value *ntree* = 100.

The artificial neural network is a fully connected feed-forward network with a single hidden layer. We use a fixed number of units between 11 and 19 in the input layer depending on the number of features of the model and a single unit with a sigmoid activation function for binary classification as the output layer. We optimized the number of units in the hidden layer as a hyperparameter (*size*) for every model in a range between [1,10] divided into steps of 1 (see Supplementary Materials for details).

In addition, all models were evaluated using three separate 10-fold cross-validations as the resampling scheme and were trained to optimize the F1 score. The final F1 score for each model is averaged over the resamples.

Classifications were assessed using sensitivity, specificity, positive predictive value (PPV), negative predictive value (NPV), F1 score, accuracy, area under the receiver operating characteristics (AUROC) and precision-recall curve (AUCPR). For AUROC and AUCPR, the 95% confidence interval was calculated (see Supplementary Materials for formulas and for detailed classification performances regarding the different models).

Area under the precision–recall curve is known to be more informative for class-imbalanced predictive tasks [32], as it is more sensitive to changes in the number of false-positive predictions. Comparison between AUROC was calculated according to DeLong et al. [33].

Analyses were implemented using R Studio (version 1.2.5001), the R Software (version 3.6.1) [34] and the following packages: *limma* [35] for plots, *rio* [36], *plyr* [37], *nlme* [38], *tidyverse* bundle [39], *pROC* [40], *ROCR* [41] for data management, data analysis and functional programming and *caret* [42] for all ML models. Graphs were generated by GraphPad Prism (version 8.4.2).

## 3. Results

The study cohort comprises 785 cases, with an average age of 75 years, the majority of individuals were male (61%), and 95% and 49% of the patients had at least one or three severe disease(s) of the Charlson comorbidity index, respectively. Most patients were hospitalized due to cardiovascular disease (40%), gastrointestinal/liver diseases (15%) or oncology disorders (15%). The prevalence of CKD in this elderly morbid cohort was comparable to other studies that included probably less morbid non-hospitalized patients ([43,44]). The prevalence for patients with no known kidney disease (NKD) was lower than for CKD. NKD was associated with younger age, better kidney function and fewer co-morbidities compared to CKD ≥ III. (Table 1).

In 128 (34%) of patients, the cause of CKD ≥ III was further specified by ICD-10 billing codes. In the remaining cohort of 245 patients with CKD ≥ III, 90% suffered from diabetes mellitus II and/or hypertension. More than 33% of etiologies for CKD ≥ III had been documented only in discharge summaries (Supplementary Materials).

There was a high incidence for AKI (33.6%) and AKI recovery (27.4%) in the CKD ≥ III cohort (Supplementary Materials).

**Figure 1:**
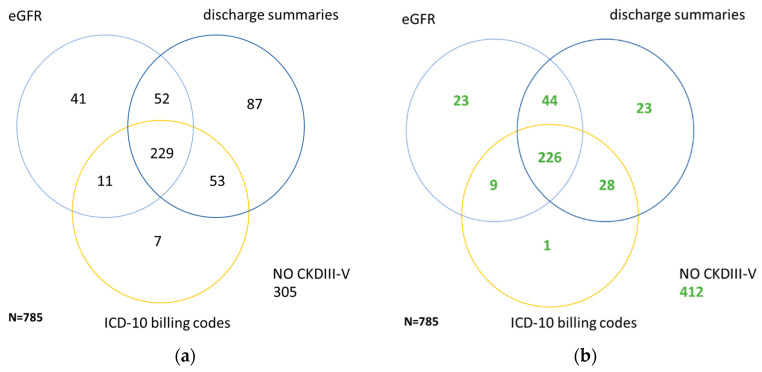
Venn diagrams comparing identification of CKD ≥ III by laboratory results (eGFR values), discharge summaries or ICD -10 billing codes within all patients (**a**) and within patients with CKD ≥ III according to reference standard (**b**). (**a**) Numbers of patients from the study cohort with CKD recognized by laboratory results (eGFR values), discharge summaries or ICD-10 billing codes. (**b**) Numbers of patients from the study cohort with CKD *correctly* recognized by laboratory results (eGFR values), discharge summaries or ICD -10 billing codes. A total of 19 patients were recognized by neither of the three formal criteria, but by manual review only.

Most patients were assigned to CKD status by discharge summaries, followed by eGFR and ICD-10 billing codes (Figure 1a). After manual review, less than 1% of the CKD cases identified by discharge summaries and eGFR and ICD-10 billing codes did not suffer from CKD III–V (Figure 1b). Patients identified by discharge summaries seemed to have a better kidney function at admission, while patients assigned to CKD by eGFR or ICD-10 billing codes had a worse kidney function compared to the reference standard. Similarly, patients identified by eGFR and discharge summaries were less morbid than patients characterized as CKD by ICD-10 billing codes, as indicated by Charlson morbidity categories (Table 2). Of note, 19 patients were identified by manual review only, while each of the three formal criteria failed.

**Figure 2:**
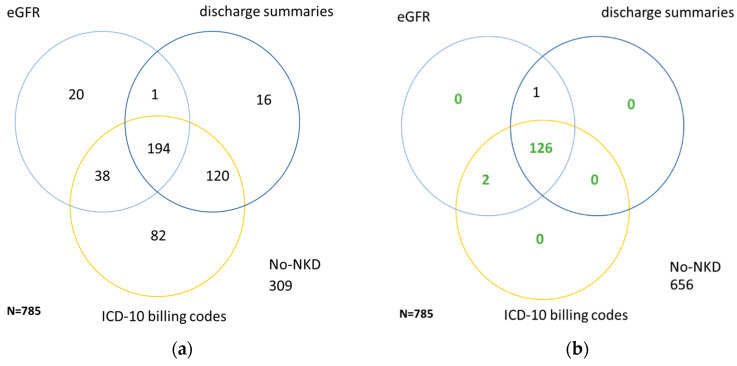
Venn diagrams comparing identification of no known kidney disease (NKD) by laboratory results (eGFR values), discharge summaries or ICD -10 billing codes within all patients (**a**) and within patients with CKD ≥ III according to reference standard (**b**). (**a**) Numbers of patients from the study cohort with NKD recognized via the eHealth sources laboratory results (eGFR values), discharge summaries or ICD-10 billing codes. (**b**) Numbers of patients from the study cohort with NKD *correctly* recognized via laboratory results (eGFR values), discharge summaries or ICD-10 billing codes.

Similar to CKD, the patient cohort was investigated for patients with no known kidney disease (NKD). Numbers of patients assigned to NKD by laboratory values, ICD-10 billing codes or discharge summaries are depicted in Figure 2a. Comparison with the reference standard (Figure 2b) confirms 65% of the patients assigned to NKD by all three categories. Patients identified by the laboratory NKD criteria were younger, had a higher eGFR at admission and did therefore better correspond with the reference standard compared to patients assigned to NKD by discharge summaries or ICD-10 billing codes (Table 3).

Table 4 and Table 5 depict the specificities and sensitivities of the different rules applied for identification of CKD or NKD, respectively. While ICD-10 billing codes show excellent specificity for identification of CKD, the sensitivity was lower compared to discharge summaries and eGFR. Discharge summaries had a better sensitivity, but a reduced specificity compared to ICD-10 billing codes (Table 4). Using eGFR < 60 mL/min/1.73 m^2^ during the whole hospital stay results in good sensitivity and specificity. If only the first eGFR at admission or the last eGFR measurement at discharge were used, overall performance (AUROC) did only minimally change compared to the original rule.

Regarding NKD, ICD-10 billing codes, discharge summaries and creatinine blood values, at admission, at discharge and during hospital stay, have all excellent sensitivity. However, acceptable specificity (>80%) was achieved only by using eGFR < 60 mL/min/1.73m^2^ during the whole hospital stay. However, the PPV was still low at 0.52 (Table 5).

**Figure 3:**
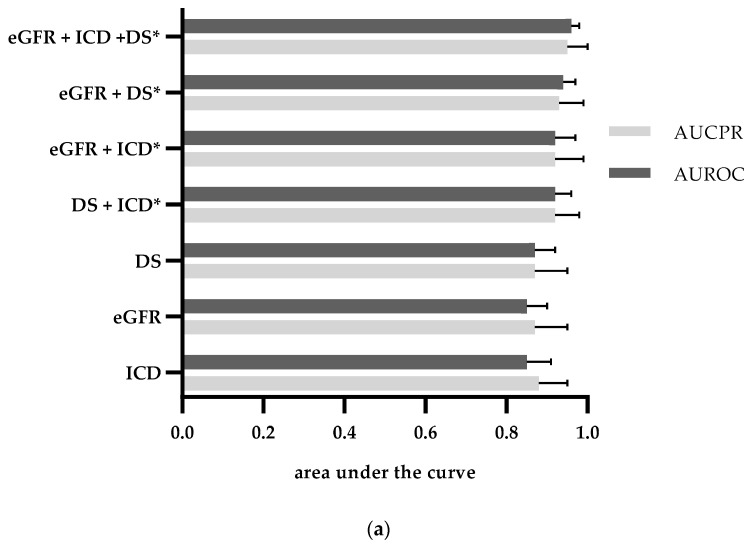

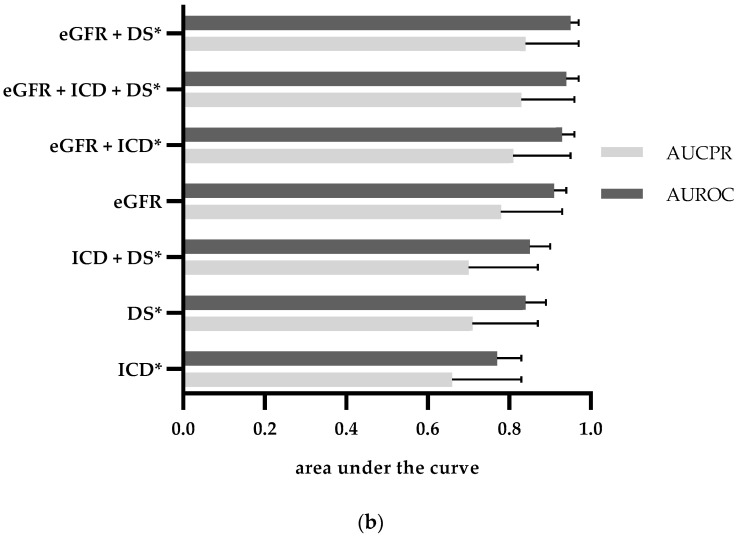
Area under the receiver operating characteristic (AUROC) and under the precision-recall curve (AUCPR) for simple categorical classifiers based on combinations of EHR components for CKD ≥ III (**a**) and NKD (**b**) on the test dataset. eGFR values = “eGFR”, discharge summaries = “DS” and ICD-10 billing codes = “ICD”. For the complete list of all combinations, see Supplementary Materials. Logistic regression was calculated on the training dataset. Performance is calculated on the test dataset (n = 156). * Indicates *p* < 0.05 for difference in AUROC compared to eGFR.

Combining laboratory measurements with discharge summaries and ICD-10 billing codes using logistic regression developed in a training dataset resulted in a better overall performance for identification of CKD (AUROC: 0.96[0.93–0.98]) or NKD (AUROC: 0.94[0.91–0.97]) in the test dataset compared to estimated glomerular filtration rate (eGFR) values (CKD: AUROC 0.85[0.79–0.90]; NKD: AUROC 0.91[0.87–0.94]), discharge summaries (CKD: AUROC 0.87[0.82–0.92], NKD: AUROC 0.84[0.79–0.89]) or ICD-10 billing codes (CKD: AUROC 0.85[0.80–0.91], NKD: AUROC 0.77[0.72–0.83) alone (Figure 3 and Supplementary Materials). Interestingly, the combination of all three categories, however, did not (NKD) or only minimally (CKD ≥ III) increase the performance in comparison with the combination of laboratory results and discharge summaries (CKD: AUROC 0.94[0.9–0.97]; NKD: AUROC 0.95[0.92–0.97]).

In NKD, AUROC values were quite high. However, AUCPR values that include sensitivity and PPV were lower. It is therefore helpful to include several parameters, e.g., AUROC and AUCPR for assessing test performance, particularly in imbalanced data [32].

To further improve performance for correct assignment of patients to CKD ≥ III or NKD, we developed a logistic regression and three ML models using (1) all data from the index hospital stay including laboratory values with incidence of AKI and AKI recovery including staging, demographics, ICD-billing codes and ICDs from discharge summaries; (2) laboratory values and demographics from the index hospital stay; (3) and (4) in addition to (1) or (2) includes laboratory values from previous hospital stays, respectively (for a detailed listing of variables, see Supplementary Materials).

**Figure 4:**
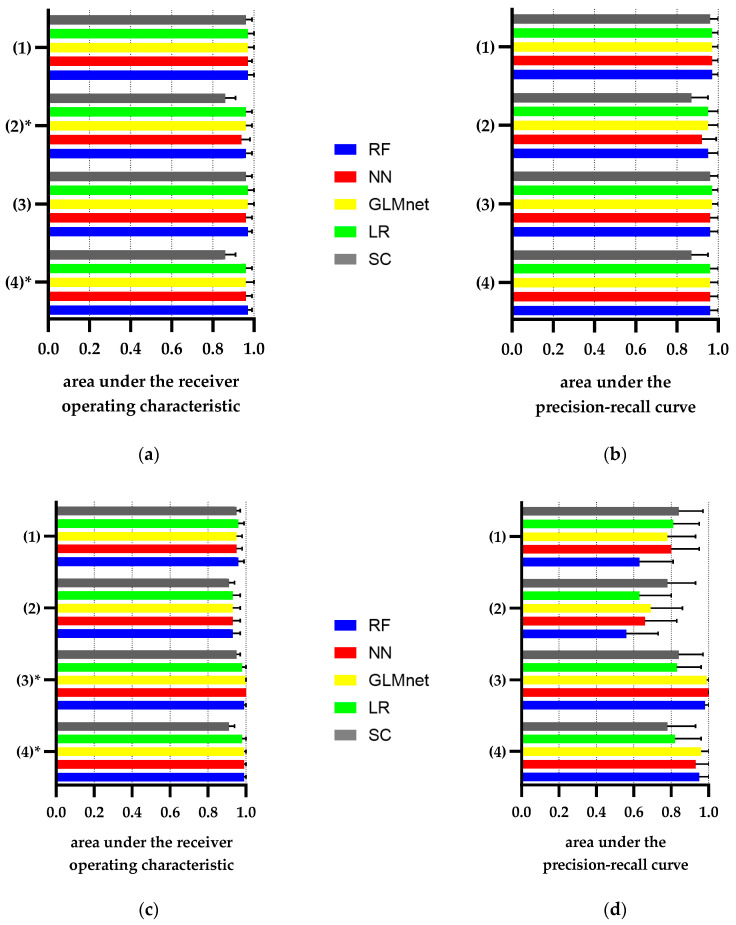
AUROC (**a**,**c**) and AUCPR (**b**,**d**) of the simple categorical classifier and of models calculated from logistic regression and the three ML methods for identification of CKD (**a**,**b**) and NKD (**c**,**d**) in different scenarios of data availability. (**a**) AUROC and (**b**) AUCPR for identification of CKD ≥ III; (**c**) AUROC and (d) AUCPR for identification of NKD. SC = simple categorical classifier, LR = logistic regression, GLMnet = generalized linear machine network, RF = random forest, NN = Artificial Neuronal Network. N = 156 patients (test dataset). Scenarios: (1) All data from the index hospital stay including laboratory values, demographics, ICD-billing codes and ICDs from discharge summaries; (2) laboratory values and demographics from the index hospital stay; (3) and (4) includes, in addition to (1) or (2), laboratory values from previous hospital stays, respectively. * Indicates *p* < 0.05 for difference in AUROC between SC and all other models.

Figure 4 shows the AUROCs and AUCPRs of the respective best logistic regression (LR) and best different ML models for identification of CKD ≥ III and NKD compared to the best simple categorical classifier for each scenario. In general, AUROCs of LR and of the different ML models were only slightly different between each other (see Supplementary Materials for more details).

For identification of CKD ≥ III, the AUROCs of the LR and machine learning models were not significantly better in scenario 1 (LR/ML: 0.97[0.95–1.00]) and scenario 3 (LR/ML: 0.97[0.94–1.00) compared to the simple classifier in scenario 1 and 3 (0.96[0.94–0.99]), respectively. AUROCs of the LR and ML models significantly (*p* < 0.05) improved in scenario 2 (LR/ML: 0.96[0.92–0.99) and scenario 4 (LR: 0.96[0.93–0.99]/ML 0.97[0.94–0.99]) compared to the simple classifier in scenario 2 and 4 (0.86[0.81–0.91]), respectively. In scenarios 2 and 4, data were restricted to laboratory values alone.

For identification of NKD, AUROCs of the LR and ML models significantly (*p* < 0.05) improved in scenario 3 (LR: 0.98[0.96–1.00]/ML: 1.00[1.00–1.00]) and scenario 4 (LR: 0.98[0.96–1.00]/ML: 0.99[0.98–1.00]) compared to the simple classifier in scenario 3 (0.95[0.92–0.97]) and scenario 4 (0.91[0.87–0.94]), respectively (Figure 4c). In scenarios 3 and 4, data from previous hospital stays were included. AUCPRs of the logistic regression and ML models for identification of NKD also improved in scenarios 3 and 4 compared to the simple classifier (Figure 4d, see Supplementary Materials for more details). AUROCs of LR and ML models slightly improved in scenario 1 (LR/ML: 0.96[0.93–0.99]) and scenario 2 (LR/ML: 0.93[0.89–0.97]) compared to the simple classifier in scenario 1 (0.95[0.92–0.97]) and scenario 2 (0.91[0.87–0.94]), respectively (Figure 4c). However, AUCPR of LR and ML models decreased in scenario 1 and 2 compared to the simple classifier. 

In conclusion, the best LR and ML models slightly improved AUROCs for identification of CKD ≥ III and NKD compared to the best simple categorical classifier in each scenario. However, we observed a significant improvement by models compared to the simple classifier for CKD > III only in scenarios 2 and 4 and for NKD only in scenarios 3 and 4.

## 4. Discussion

The results of our study demonstrate that laboratory values have the best performance for identifying CKD ≥ III and NKD from EHRs compared to discharge summaries and ICD-10 billing codes in an elderly multimorbid cohort of hospitalized patients. Combining classifiers based on laboratory values (creatinine/eGFR), ICD-10 billing codes or ICD-10 codes extracted from discharge summaries outperformed each component alone for identification of CKD ≥ III and NKD. Classification could be further improved by calculation of logistic regression and ML models if data were restricted to laboratory values (CKD ≥ III) or if additional values from previous hospital stays were added (NKD).

Although each of the mentioned EHR components have been investigated before, we could demonstrate the extent to which the classification is improved by combining laboratory values with ICD-10 billing codes and discharge summaries. Furthermore, we are the first, to our knowledge, to describe classification performance for NKD.

The good sensitivity and specificity of laboratory values for the identification of CKD ≥ III and NKD can be explained by the fact that both entities are mainly defined by blood creatinine and eGFR values [3,26]. However, many epidemiological studies and clinical trials have utilized ICD-10 billing codes for defining CKD status [4]—more than 50% of cardiovascular trials do not report eGFR measurement in respective study populations [45].

Previous studies have demonstrated a high specificity of billing codes. However, many CKD patients will be overlooked by using billing codes alone and the identified cohort is biased towards more advanced CKD stages with higher creatinine values [5,46,47]. These results have been replicated and confirmed in the current study. A sensitivity of 75% indicates that approximately one-quarter of patients with advanced CKD ≥ III had been missed by ICD-10 billing codes. Patients recognized by ICD-10 billing codes had a lower eGFR and showed a higher morbidity in comparison to the reference standard.

However, the sensitivity of ICD-10 billing codes was much better in our study than in a recent study by Diamantidis et al. who reported a very low sensitivity of ICD-10 billing codes for recognizing CKD > III [43]. The discrepancy might be explained by differences in the patient cohorts as the latter study included non-hospitalized patients.

Gomez-Salgado et al., in contrast, recently showed good correlation between ICD-10 billing codes and researchers’ judgment based on clinical documentation [48]. A possible explanation for the conflicting results between our study and Gomez-Salgado et al. could be the extent to which laboratory values were considered for identification of CKD.

Our study also confirms previous findings of slight under-documentation of CKD using discharge summaries [49]. Indeed approximately 20% of patients with advanced CKD ≥ III were not identified by discharge summaries. However, in line with the study of Singh et al., we could also show that the sensitivity of discharge summaries is higher than the sensitivity of billing codes for CKD [9]. The reduced specificity of discharge summaries could be explained by the fact that many patients with CKD stage I and II were counted as CKD ≥ III. Differing definitions for chronic kidney disease might also be the reason why a recent study by Hernandez-Boussard et al. observed a better accuracy for unstructured discharge summaries for recognizing CKD compared to our study [50]. Other possible explanations are different information sources and a different study cohort.

In a study by Nadkarni et al., an algorithm was developed and evaluated to identify patients with CKD Stage III caused by hypertension or diabetes, using structured and unstructured information from EHRs [51]. The algorithm based on keywords from medical notes and laboratory values outperformed phenotyping by ICD-10 billing codes by a margin. These results resonate with the outcome of our study that included advanced CKD from any cause in hospitalized patients.

Missing previous health records is a common problem in clinical studies and might affect correct identification of diseases [52]. However, in contrast to the identification of patients with diabetes mellitus [53], we can demonstrate good F1 score (>0.8), although using datasets restricted to the current hospital stay for simple classifiers. For CKD ≥ III, ML models based on laboratory values alone had a similar AUROC as the simple categorical classifiers including discharge summaries and ICD-10 billing codes. This indicates that ML models might be able to—at least partly—compensate for missing information.

The results of our study are encouraging, not only for stratification of patients for clinical and epidemiological studies, but also in the context of, e.g., Healthcare-Integrated Biobanking, where automated classifiers based on minimal clinical information are of great importance for early selection of samples of specific disease entities.

Structured information such as laboratory values and billing codes are often readily available. Results from our study show that a PPV of 0.77, 0.82 or 0.91 can be achieved for the identification of CKD by using eGFR values at admission, at discharge or from the complete hospital stay, respectively. This is in line with other studies demonstrating that a single measurement of eGFR might overestimate the number of CKD cases [54]. The slightly higher PPV when using eGFR values at discharge compared to admission can be explained by the fact that interfering acute kidney injury is more likely to be present at admission than after a successful treatment at discharge.

Suboptimal PPV values associated with false classification can significantly impact the phenotyping process and thus might cause severe bias in the outcomes of subsequent studies. Consequently, there is a need for further optimization of CKD and NKD classification.

Wei et al. combined different sources of information (primary notes, medication and billing codes) to improve phenotyping based on EHR for several chronic diseases (not CKD though) and demonstrated that PPV and F1 score can be increased by combining different information sources [55]. Results from Wei et al. can be confirmed in our study in relation to CKD and NKD with the caveat that eGFR should be included in any combination.

The addition of discharge summaries and/or ICD-10 billing codes to laboratory values not only increases the performance of correct identification of CKD ≥ III but also helped to further specify the cause of the disease in at least one-third of the cohort. There were more etiologies for CKD in the discharge summaries compared to the ICD-10 billing codes.

Another novelty of this study is that, to the best of the authors’ knowledge, for the first time the entity of NKD (no known kidney disease) was investigated using EHRs. Identifying NKD is a challenging task because ICD-10 billing codes and discharge summaries are designed to describe the presence of illness rather than its absence. However, the question of NKD might be of particular interest for scientific reasons. The validity of association studies and clinical trials depends on the correct assignment of co-morbidities. If large cohorts of CKD patients are counted as NKD, studies might be biased and results might thus be flawed. Our study demonstrates that single EHR sources had low PPV and AUCPR for NKD assignment. Combining laboratory values with discharge summaries improved PPV and AUCPR. Interestingly, the further addition of ICD-10 billing codes to this combination did not result in a further improvement of PPV and AUCPR. Future epidemiological studies should take these results in consideration for classification of NKD.

Finally, we demonstrated that logistic regression and ML algorithms have the potential to improve recognition of CKD ≥ III and NKD, particularly in certain scenarios of data availability. This might be helpful for the development of clinical decision support systems (CDSS) in the near future that ultimately will allow clinicians and researches almost instantly to evaluate the chronic kidney status of patients.

Direct comparison with other studies applying ML strategies for the detection of CKD is hampered due to different definitions of CKD, different patient cohorts and data variables used. Almansour et al. described an Artificial Neural Network with an accuracy of more than 99% [20]. Salekin et al. used the same cohort and reduced the number of variables down to 12 and achieved an F1 score of 99% by using a wrapper approach to identify the best subset of attributes and a random forest classifier [56]. However, both studies rely on the same data source comprising 24 variables of 400 patients to build a predictive model. In contrast to our study, the dataset does not include series of creatinine measurements or information from discharge summaries or ICD-10 billing codes about CKD. Rashidian et al. used laboratory values, demographics and ICD-10 billing codes to identify patients with CKD achieving a F1 score of approximately 0.8 [57]. In our study, AUROC and AUCPR for identification of CKD from ML algorithms surpassed 0.95 in all scenarios of unrestricted or restricted data availability. One reason for these differences could be that the study by Rashidian et al. did not use discharge letters as source of information. As mentioned before, in our study discharge summaries can add valuable information to the classification process. This is also reflected by the result that ML algorithms did not significantly improve performance of CKD ≥ III identification (AUROC 0.97) compared to a simple classifier based on laboratory values, discharge summaries and ICD-10 billing codes (AUROC 0.96).

The ML algorithms used in our study failed to outperform rule-based classifiers for identification of NKD if data were restricted to the index hospital stay: although AUROC is (non-significantly) increasing, PPV is declining and thus superiority of the models has to be rejected. An explanation for this result could be that the correct assignment of NKD mainly depends on the availability of the complete dataset. Additionally, we cannot exclude that the low prevalence of NKD in our morbid patient cohort affected the efficacy of ML strategies.

To the best of our knowledge, this is the first study trying to detect specifically CKD Stage ≥ III and NKD by ML methods. Therefore, it is mandatory that the proof-of-concept presented here needs further elaboration in larger independent patient cohorts.

The strength of the study is the comprehensive dataset including discharge summaries of the index hospital stay and laboratory values with a reviewed reference standard.

Several limitations need to be acknowledged. The patient cohort included in the study was quite morbid and not representative of a general hospital population or, even more so, an outpatient population. Therefore, the extent of improvement by combining different information sources needs to be prospectively validated in other independent cohorts.

The Averbis Health Discovery software tool was used for the extraction of information attributes from discharge summaries that have been predefined by the authors. The use of natural language processing (NLP) methods for information extraction and automated feature selection could have resulted in an increased performance of the data extraction method.

Similarly, the total number of patients was rather small for training ML classifiers. We may guess that, in a larger patient cohort, the performance of the different models might further increase. However, the scope of the present study was to demonstrate the feasibility and potential of using eHealth sources and ML models to improve phenotyping of CKD and NKD.

The models presented in this manuscript focus on the detection of advanced CKD (Stage III or higher) or on the absence of kidney disease. Patients with mild CKD (Stage I and II) are not taken into consideration although the correct identification of this group might be important for clinical treatment and research purpose. Future studies with larger patient cohorts might be able to develop more granular models differentiating between mild and advanced CKD.

Another limitation is that neither a single rule nor a combination of them achieved a sensitivity for identification of CKD ≥ III of 100%. This could be explained by the fact that most patients were treated primarily for non-nephrological reasons during the index hospital stay and thus CKD was not mentioned at all in the current discharge summaries or by the ICD-10 billing codes, although they had a documented eGFR < 60 mL/min/1.73m^2^ for a period longer than 90 days.

Furthermore, data included in the analysis were incomplete, since laboratory results from primary care or other institutions (for example, from general practitioners or other hospitals) were not available. Most importantly albuminuria was available in less than 5% of the whole cohort and could therefore not included in the analysis.

Missing data, however, reflects “real-world” conditions. Missing data can be, at least partly, compensated for—as shown in our study—by the extraction of unstructured information from the discharge summaries that usually contain a multitude of pre-existing health data from other healthcare providers.

## 5. Conclusions

In summary, combining laboratory results (creatinine and eGFR) with discharge summaries and ICD-10 billing codes had the best performance in a simple categorical classifier for phenotyping of CKD ≥ III and NKD. Logistic regression or ML models had the potential to further improve the correct identification of CKD ≥ III if only laboratory values were used and of NKD if data from previous hospital stays were included into models.

## Figures and Tables

**Table 1:** Epidemiological Characteristics from all Individuals and from Individuals with CKD ≥ III or NKD Identified by the Reference Standard, Respectively.

Characteristics	Cohort (*n* = 785)	CKD ≥ III (*n* = 373)	NKD (*n* = 129)
Age, years, mean [SD]	74.6 [12.2]	77.9 [10]	68.4 [13.7]
Sex, male	476 (60.6%)	215 (57.6%)	79 (61.2%)
eGFR at admission, median, [quartiles], mL/min/1.73 m^2^	(*n* = 780) ^1^ 49.6 [28.6–77.3]	(*n* = 372) ^1^ 28.9 [18.1–41.8]	88.6 [78.5–99.6]
	(*n* = 748)		
Charlson morbidity category ≥1	711 (95.3%)	366 (98.1%)	113 (87.6%)
≥3	387 (49.3%)	224 (60.1%)	36 (27.9%)
Median	2	3	2
Myocardial infarction	128 (16.3%)	75 (20.1%)	11 (8.5%)
Chronic heart failure	419 (54.4)	247 (66.2%)	33 (25.6%)
Peripheral vascular disease	131 (16.7%)	75 (20.1%)	17 (13.2%)
Cerebrovascular disease	51 (6.5%)	28 (7.5%)	7 (5.4%)
Dementia	31 (3.9%)	18 (4.8%)	4 (3.1%)
Chronic pulmonary disease	183 (23.3%)	73 (16.9%)	23 (17.8%)
Rheumatic diseases	13 (1.7%)	4 (1.1%)	3 (2.3%)
Peptic ulcer disease	21 (2.7%)	11 (2.9%)	1 (0.8%)
Hemiplegia or paraplegia	29 (3.7%)	8 (2.1%)	6 (4.7%)
Liver disease	137 (17.5%)	44 (11.8%)	35 (25.1%)
Diabetes mellitus	332 (42.3%)	152 (40.7%)	51 (39.5%)
Any malignancy	137 (17.5%)	32 (8.6%)	38 (29.5%)
Hypertension	567 (72.3%)	270 (72.4%)	93 (72.1%)
Major cause for admission			
Infectious diseases	58 (7.4%)	28 (7.5%)	6 (4.7%)
Oncology disorders	119 (15.2%)	30 (8.0%)	34 (26.4%)
Cardiovascular	315 (40.1%)	192 (51.5%)	40 (31.0%)
Diseases			
Pulmonary diseases	82 (10.4%)	25 (6.7%)	12 (9.3%)
Gastrointestinal	118 (15.0%)	35 (9.4%)	27 (20.9%)
and liver diseases			
Kidney diseases	47 (6.0%)	36 (9.7%)	2 (1.6%)
other	46 (5.9%)	27 (7.2%)	8 (6.2%)

^1^ eGFR at admission could not be calculated for all individuals because creatinine was massively interfered with by bilirubin or hemoglobin at admission.

**Table 2:** Epidemiological characteristics from patients with CKD identified by reference standard or recognized by laboratory results (eGFR values), discharge summaries or ICD-10 billing codes.

Characteristics	Reference Standard (*n* = 373)	eGFR (*n* = 333)	Discharge Summaries (*n* = 421)	ICD-10 Billing Codes (*n* = 300)
Age, years, mean [SD]	77.9 [10]	78.0 [9.7]	76.4 [10.9]	77.2 [10.3]
Sex, male	215 (57.6%)	189 (56.8%)	258 (61.3%)	182 (60.7%)
eGFR at admission, median, [quartiles], mL/min/1.73 m^2^	(*n* = 372) ^1^ 28.9 [18.1–41.8]	26.8 [17.5–39.4]	(*n* = 420) ^1^ 32.9 [19.6–50]	25.7 [15.2–39.6]
Charlson morbidity category ≥1	366 (98.1%)	326 (97.9%)	413 (98.1%)	297 (99%)
≥3	224 (60.1%)	198 (59.5%)	257 (61.1%)	220 (73.3%)
Median	3	3	3	3

^1^ eGFR could not be calculated for all individuals because creatinine was massively interfered with by bilirubin or hemoglobin at admission.

**Table 3:** Epidemiological characteristics from patients with NKD identified by reference standard or recognized by sources laboratory results (eGFR values), discharge summaries or ICD-10 billing codes.

Chracteristics	Reference Standard (*n* = 129)	eGFR (*n* = 253)	Discharge Summaries (*n* = 334)	ICD-10 Billing Codes (*n* = 437)
Age, years, mean [SD]	68.4 [13.7]	69.3 [13.3]	72.9 [13.3]	73.3 [13.0]
Sex, male	79 (61.2%)	161 (63.6%)	196 (58.7%)	265 (60.6%)
eGFR at admission, median, [quartiles], mL/min/1.73m^2^	88.6 [78.6–99.3]	84.5 [75.7–96.2]	76.0 *^,1^ [53.8–89.5]	69.9 *^,2^ [50.0–87.7]
Charlson morbidity score ≥1	113 (87.6%)	232 (91.7%)	308 (92.2%)	403 (92.2%)
≥3	36 (27.9%)	91 (36.0%)	116 (34.7%)	145 (33.2%)
Median	2	2	2	2

* eGFR could not be calculated for all individuals because creatinine was massively interfered with by bilirubin or hemoglobin at admission. ^1^ *n* = 331; ^2^ *n* = 434.

**Table 4:** Performance of different rules for identification of patients with CKD compared to the reference standard.

Category	Sensitivity	Specificity	PPV	NPV	AUROC (CI)	AUCPR (CI)
ICD-10 billing codes	0.71	0.91	0.88	0.78	0.81 (0.78–0.84)	0.86 (0.83–0.90)
Discharge summary	0.86	0.76	0.76	0.86	0.81 (0.78–0.84)	0.84 (0.81–0.88)
eGFR <60 mL/min/1.73 m^2^ during Index hospital stay	0.81	0.92	0.91	0.84	0.87 (0.84–0.90)	0.90 (0.87–0.93)
eGFR_at_admission <60 mL/min/1.73 m^2^	0.96	0.75	0.77	0.95	0.85 (0.83–0.87)	0.88 (0.84–0.91)
eGFR_at_discharge <60 mL/min/1.73 m^2^	0.91	0.82	0.82	0.91	0.86 (0.84–0.89)	0.89 (0.85–0.92)

**Table 5:** Performance of different rules for identification of patients with NKD compared to the reference standard.

Category	Sensitivity	Specificity	PPV	NPV	AUROC (CI)	AUPR (CI)
ICD-10 billing codes	0.99	0.53	0.29	1	0.76	0.64
					(0.74–0.78)	(0.56–0.73)
Discharge summary	0.98	0.68	0.38	1	0.83	0.68
					(0.81–0.86)	(0.60–0.76)
eGFR ≥ 60 mL/min/1.73m^2^ during Index hospital stay	1.00	0.82	0.52	1	0.91 (0.89–0.92)	0.75 (0.68–0.83)
eGFR_at_admission ≥ 60 mL/min/1.73 m^2^	1.00	0.71	0.41	1.00	0.86 (0.84–0.87)	0.70 (0.62–0.78)
eGFR_at_discharge ≥ 60 mL/min/1.73 m^2^	1.00	0.64	0.35	1.00	0.82 (0.80–0.84)	0.68 (0.59–0.76)

## Supplementary Materials

Supplementary Materials are available online. https://www.mdpi.com/2077-0383/9/9/2955/s1, Table S1: Characteristics of the study cohort; Additional characteristics of the study cohort; Table S2: ICD-10 billing codes for definition of CKD; Table S3: ICD-10 billing codes for exclusion of NKD; Table S4: detailed performance characteristics for combinations of simple classifiers for identification of CKD and NKD; Table S5: Detailed AUC-ROC and -PR for combinations of different classifiers for identification of CKD and NKD; Table S6: Cause for CKD in the CKD>III cohort; detailed cause for CKD ≥ III and source of information; Table S7: Incidence of AKI and AKI Recovery in the complete study cohort with creati-nine values (n=780) and in CKD>III cohort with creatinine values (n=372); Table S8: Source of information for etiologies of CKD>III; Table S9: Distribution of true positives and true negatives for CKD and NKD, in the training and test datasets; Table S10: Detailed performance characteristics for combinations of different classifiers for identification of CKD and NKD; Table S11: Detailed AUC-ROC and -PR for combinations of different classifiers for identification of CKD and NKD; Table S12: Detailed performance characteristics for different generalized linear model networks for identification of CKD and NKD; Table S13: Detailed AUC-ROC and -PR for different generalized linear model networks for identification of CKD and NKD; Table S14: Detailed performance characteristics for different gen-eralized linear model networks for identification of CKD and NKD; Table S15: Detailed AUC-ROC and -PR for different generalized linear model networks for identification of CKD and NKD; Table S16: Detailed performance characteristics for different random forest models for identification of CKD and NKD; Table S17: Detailed AUC-ROC and -PR for different random forest models for identification of CKD and NKD; Table S18: Detailed performance characteristics for different random forest models for identification of CKD and NKD; Table S19: Detailed AUC-ROC and -PR for different random forest models for identification of CKD and NKD; Table S20: Detailed performance characteristics for different neural networks models for identification of CKD and NKD; Table S21: Detailed AUC-ROC and -PR for for different neural networks models for identification of CKD and NKD; Table S22: Detailed performance characteristics for different neural networks models for identification of CKD and NKD; Table S23: Detailed AUC-ROC and -PR for for different neural networks models for identification of CKD and NKD; Table S24: Detailed performance characteristics for different generalized linear mod-els for identification of CKD and NKD; Table S25: Detailed AUC-ROC and -PR for for different generalized linear models for identification of CKD and NKD; Table S26: Detailed performance characteristics for different generalized linear models for identification of CKD and NKD; Table S27: Detailed AUC-ROC and -PR for for different generalized linear models for identification of CKD and NKD; Table S28: Detailed hyperparameters of different machine learning models; Table S29: Detailed hyperparameters of different machine learning models.
