# Supplementary material for: Optimized Identification of Advanced Chronic Kidney Disease and Absence of Kidney Disease by Combining Different Electronic Health Data Resources and by Applying Machine Learning Strategies
Source: J Clin Med. 2020 Sep 12;9(9):2955. doi: 10.3390/jcm9092955 (PMC7563476; doi:10.3390/jcm9092955)
Supplement: Supplementary file 1 [file jcm-09-02955-s001.pdf]

Supplemental 1: Characteristics of the study cohort

Chracteristics & outcome	Total (n=785)
Age, years, mean[SD]	74.6 [12.2]
Sex, male n (%)	476 (60.6%)
eGFR at admission,	(n=780)
median [I quartile - III quartile]	49.6 [28.6 - 77.3]
eGFR < 60ml/min n (%)	462 (59.2%)
eGFR at discharge,	(n=780)
median [I quartile - III quartile]	57.4 [53.1 - 83.3]
eGFR < 60ml/min n (%)	412 (52.8%)
eGFR over Index,	(n=780)
mean of means, median [I quartile - III quartile]	54.4 [33.6 - 80.1]
mean of eGFR over Index < 60ml/min, n (%)	436 (55.9%)
all eGFR < 60 ml/min throughout Index, n (%)	333 (42.7%)
Charlson Morbidity score, n (%)	748 (95.3%)
0 (0)	37
1 (1)	149
2 (2)	212
3 (ab 3)	387

median	2

Supplemental 2: ICD-10 codes for definition of CKD

- *N18.3-N18.9, Z99.2, N19, N00-N08, N10-N16, N25, N26, N28, N29, I12, I13, Q61, Z49, Z94.0*

Supplemental 3: ICD-10 codes for exclusion of NKD

- *A18.1, A52.7, B52.0, C64, C68.9, D30.0, D41.0-D41.2, D59.3, E10.2x, E10.6x, E11.2x, E11.6x, E13.2x, E14.2x, E74.8, K76.7, M10.3x, N17.x, N18.1-N18.2, O10.4, O12.x, O26.81, O90.4, Q60.x, R31, R80, R94.4, Z99.2*

Supplemental 4: Detailed performance characteristics for combinations of different classifiers for identification of CKD and NKD. All patients included.

**CKD**	**Sensitivity**	**Specificity**	**PPV**	**NPV**	**F1-score**	**Accuracy**
	= TP/(TP+FN)	= TN/(TN+FP)	= TP/(TP+FP)	= TN/(TN+FN)	= 2*(PPV*Sensitivity)/(PPV+Sensitivity)	= (TP+TN)/(TP+FP+TN+FN)
ICD + DS	0.89	0.74	0.75	0.88	0.82	0.81
DS	0.86	0.76	0.76	0.86	0.81	0.81
ICD	0.71	0.91	0.88	0.78	0.78	0.82
eGFR_admission	0.96	0.75	0.77	0.95	0.86	0.85
eGFR_admission + ICD	0.97	0.71	0.75	0.96	0.84	0.83

eGFR_admission + ICD + DS	0.99	0.59	0.68	0.98	0.81	0.78
eGFR_admission + DS	0.99	0.59	0.69	0.98	0.81	0.78
eGFR_discharge	0.91	0.82	0.82	0.91	0.86	0.86
eGFR_discharge + ICD	0.93	0.77	0.79	0.93	0.85	0.85
eGFR_discharge + ICD + DS	0.98	0.64	0.71	0.97	0.82	0.8
eGFR_discharge + DS	0.98	0.64	0.71	0.97	0.82	0.8
eGFR	0.81	0.92	0.91	0.84	0.86	0.87
eGFR + ICD	0.89	0.85	0.84	0.89	0.86	0.87
eGFR + ICD + DS	0.95	0.69	0.74	0.94	0.83	0.82
eGFR + DS	0.95	0.71	0.75	0.94	0.83	0.82
**NKD**						
ICD	0.99	0.53	0.29	1	0.45	0.61
DS	0.98	0.68	0.38	1	0.55	0.73
ICD + DS	0.98	0.71	0.4	0.99	0.57	0.75
eGFR_admission	1	0.71	0.41	1	0.58	0.76
eGFR_admission + DS	0.98	0.84	0.55	1	0.71	0.87
eGFR_admission + ICD	0.99	0.76	0.45	1	0.62	0.8
eGFR_admission + ICD + DS	0.98	0.85	0.56	0.99	0.71	0.87
eGFR_discharge	1	0.64	0.35	1	0.52	0.7
eGFR_discharge + DS	0.98	0.8	0.5	1	0.66	0.83

eGFR_discharge + ICD	0.99	0.72	0.41	1	0.58	0.76
eGFR_discharge + ICD + DS	0.98	0.81	0.5	0.99	0.66	0.84
eGFR	1	0.81	0.51	1	0.68	0.84
eGFR + DS	0.98	0.9	0.65	1	0.78	0.91
eGFR + ICD	0.99	0.84	0.55	1	0.71	0.87
eGFR + ICD + DS	0.98	0.9	0.65	0.99	0.78	0.91

Supplemental 5: Detailed AUC-ROC and -PR for combinations of different classifiers for identification of CKD and NKD. All patients included.

**CKD**	**ROC lower bound**	**ROC**	**ROC upper bound**	**PR lower bound**	**PR**	**PR upper bound**
ICD + DS	0.85	0.87	0.9	0.85	0.89	0.92
DS	0.78	0.81	0.84	0.81	0.84	0.88
ICD	0.78	0.81	0.84	0.83	0.86	0.9
eGFR_admission	0.83	0.85	0.87	0.84	0.88	0.91
eGFR_admission + ICD	0.9	0.92	0.94	0.89	0.92	0.95
eGFR_admission + ICD + DS	0.92	0.94	0.95	0.91	0.93	0.96
eGFR_admission + DS	0.9	0.92	0.94	0.89	0.92	0.95
eGFR_discharge	0.84	0.86	0.89	0.85	0.89	0.92

eGFR_discharge + ICD	0.9	0.92	0.94	0.9	0.92	0.95
eGFR_discharge + ICD + DS	0.92	0.94	0.95	0.91	0.94	0.96
eGFR_discharge + DS	0.91	0.93	0.94	0.9	0.93	0.95
eGFR	0.84	0.87	0.89	0.87	0.9	0.93
eGFR + ICD	0.89	0.91	0.93	0.91	0.94	0.96
eGFR + ICD + DS	0.92	0.94	0.95	0.92	0.95	0.97
eGFR + DS	0.91	0.93	0.94	0.91	0.94	0.96
**NKD**						
ICD	0.74	0.76	0.78	0.56	0.64	0.73
DS	0.81	0.83	0.86	0.6	0.68	0.76
ICD + DS	0.83	0.85	0.87	0.61	0.69	0.77
eGFR_admission	0.84	0.86	0.87	0.62	0.7	0.78
eGFR_admission + DS	0.91	0.92	0.93	0.7	0.77	0.85
eGFR_admission + ICD	0.86	0.88	0.9	0.65	0.72	0.8
eGFR_admission + ICD + DS	0.91	0.92	0.94	0.7	0.77	0.85
eGFR_discharge	0.8	0.82	0.84	0.59	0.68	0.76
eGFR_discharge + DS	0.88	0.9	0.92	0.67	0.74	0.82
eGFR_discharge + ICD	0.84	0.86	0.88	0.62	0.7	0.78
eGFR_discharge + ICD + DS	0.89	0.9	0.92	0.67	0.74	0.82
eGFR	0.89	0.91	0.92	0.68	0.75	0.83
eGFR + DS	0.93	0.95	0.96	0.76	0.82	0.89
eGFR + ICD	0.91	0.92	0.93	0.7	0.77	0.85

eGFR + ICD + DS	0.93	0.95	0.96	0.75	0.82	0.89

Supplemental 6: Cause for CKD in the CKD>III cohort (n=373)

			**number (percentage)**
**Specification of renal disease**			128 (34.3%)
	hypertensive nephropathy		67 (52.3%)
	glomerular disease		56 (43.7%)
	tubular disease		27 (21.0%)
	malignancy		11 (8.59%)
	transplantation		22 (17.1%)
	cystic kidney disease		21 (16.4%)
	hepatorenal disease		5 (3.90%)
**No further specification**			245 (65.6%)
	Diabetes mellitus		194 (79.1%)
	Hypertension		127 (51.8%)
	neither disease		25 (10.2%)

Supplemental 7: Incidence of AKI and AKI Recovery in the complete study co-hort with creatinine values (n=780) and in CKD>III cohort with creatinine values (n=372):

			**Complete Cohort**	**CKD≥III cohort**
**AKI**			161 (20.6%)	114 (30.4%)
	Stage 1		103 (13.2%)	72 (19.4%)
	Stage 2		25 (3.2 %)	13 (3.5%)
	Stage 3		33 (4.2%)	29 (7.5%)

**AKI recovery**		166 (12.9%)	102 (27.5%)
	Stage 1	101 (6.7%)	61 (16.4%)
	Stage 2	52 (3.2 %)	30 (8.1%)
	Stage 3	13 (1.7%)	11 (3%)

Supplemental 8: Source of information for etiologies of CKD>III

Figure. Percentage of ICD-codes further specifying the reason for CKD≥III extracted from billing codes, discharge letters or both

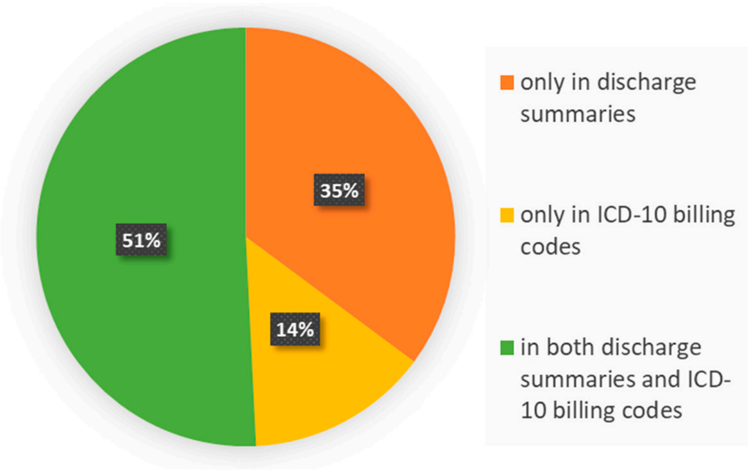

In 128 (34%) of patients ICD codes further specified the reason for CKD≥III. The color indicates the percentage specific ICD codes derived from discharge summaries alone (orange), ICD-10 billing codes alone (yellow) or from both categories (green).

Supplemental 9: Distribution of true positives and true negatives for CKD and NKD, in the training and test datasets

Classification	CKD		NKD	
	train	test	train	test
positive	303	70	110	30
negative	325	87	518	127

Supplemental 10: Detailed performance characteristics for combinations of different classifiers for identification of CKD and NKD. Only patients from test data included.

CKD	Sensitivity	Specificity	PPV	NPV	F1-score	Accuracy
	= TP/(TP+FN)	= TN/(TN+FP)	= TP/(TP+FP)	= TN/(TN+FN)	= 2*(PPV*Sensitivity)/(PPV+Sensitivity)	= (TP+TN)/(TP+FP+TN+FN)
ICD + DS	0.96	0.74	0.74	0.96	0.83	0.83
DS	0.96	0.78	0.77	0.96	0.86	0.86
ICD	0.81	0.9	0.86	0.86	0.83	0.86
eGFR_admission	0.97	0.73	0.73	0.97	0.84	0.83
eGFR_admission + ICD	0.97	0.67	0.69	0.97	0.81	0.8
eGFR_admission + ICD + DS	1	0.57	0.64	1	0.78	0.76
eGFR_admission + DS	1	0.59	0.65	1	0.79	0.77
eGFR_discharge	0.91	0.81	0.78	0.92	0.84	0.85
eGFR_discharge + ICD	0.96	0.76	0.76	0.96	0.84	0.85
eGFR_discharge + ICD + DS	1	0.65	0.69	1	0.81	0.8
eGFR_discharge + DS	1	0.65	0.69	1	0.81	0.8
eGFR	0.79	0.9	0.86	0.85	0.82	0.85
eGFR + ICD	0.93	0.82	0.8	0.94	0.86	0.87

eGFR + ICD + DS	0.99	0.69	0.71	0.98	0.83	0.82
eGFR + DS	0.99	0.72	0.73	0.98	0.84	0.83
**NKD**						
ICD	0.97	0.58	0.35	0.99	0.52	0.65
DS	0.97	0.71	0.45	0.99	0.61	0.76
ICD + DS	0.93	0.73	0.45	0.98	0.61	0.77
eGFR_admission	1	0.71	0.45	1	0.62	0.77
eGFR_admission + DS	0.97	0.84	0.59	0.99	0.73	0.87
eGFR_admission + ICD	0.97	0.79	0.52	0.99	0.67	0.82
eGFR_admission + ICD + DS	0.93	0.85	0.6	0.98	0.73	0.87
eGFR_discharge	1	0.63	0.39	1	0.56	0.7
eGFR_discharge + DS	0.97	0.8	0.54	0.99	0.69	0.83
eGFR_discharge + ICD	0.97	0.74	0.47	0.99	0.63	0.78
eGFR_discharge + ICD + DS	0.93	0.8	0.53	0.98	0.67	0.83
eGFR	1	0.82	0.57	1	0.72	0.85
eGFR + DS	0.97	0.9	0.69	0.99	0.81	0.91
eGFR + ICD	0.97	0.87	0.63	0.99	0.76	0.88
eGFR + ICD + DS	0.93	0.9	0.68	0.98	0.79	0.9

Supplemental 11: Detailed AUC-ROC and -PR for combinations of different classifiers for identification of CKD and NKD. Only patients from test data included.

**CKD**	**ROC lower bound**	**ROC**	**ROC upper bound**	**PR lower bound**	**PR**	**PR upper bound**
ICD + DS	0.88	0.92	0.96	0.85	0.92	0.98
DS	0.82	0.87	0.92	0.8	0.87	0.95
ICD	0.8	0.85	0.91	0.8	0.88	0.95
eGFR_admission	0.8	0.85	0.9	0.78	0.86	0.94
eGFR_admission + ICD	0.9	0.94	0.97	0.87	0.93	0.99
eGFR_admission + ICD + DS	0.94	0.96	0.99	0.91	0.96	1
eGFR_admission + DS	0.91	0.95	0.98	0.88	0.94	0.99
eGFR_discharge	0.81	0.86	0.91	0.79	0.87	0.95
eGFR_discharge + ICD	0.88	0.92	0.97	0.85	0.92	0.98
eGFR_discharge + ICD + DS	0.94	0.96	0.99	0.91	0.96	1
eGFR_discharge + DS	0.91	0.95	0.98	0.88	0.94	0.99
eGFR	0.79	0.85	0.9	0.79	0.87	0.95
eGFR + ICD	0.88	0.92	0.97	0.86	0.92	0.99
eGFR + ICD + DS	0.93	0.96	0.98	0.9	0.95	1
eGFR + DS	0.9	0.94	0.97	0.87	0.93	0.99
**NKD**						

ICD	0.72	0.77	0.83	0.49	0.66	0.83
DS	0.79	0.84	0.89	0.55	0.71	0.87
ICD + DS	0.8	0.85	0.9	0.54	0.7	0.87
eGFR_admission	0.82	0.86	0.9	0.57	0.73	0.89
eGFR_admission + DS	0.88	0.92	0.95	0.64	0.79	0.93
eGFR_admission + ICD	0.85	0.89	0.93	0.59	0.75	0.9
eGFR_admission + ICD + DS	0.88	0.92	0.95	0.63	0.78	0.93
eGFR_discharge	0.77	0.81	0.86	0.53	0.69	0.86
eGFR_discharge + DS	0.86	0.89	0.93	0.61	0.76	0.91
eGFR_discharge + ICD	0.82	0.86	0.9	0.56	0.72	0.88
eGFR_discharge + ICD + DS	0.85	0.89	0.93	0.59	0.75	0.9
eGFR	0.87	0.91	0.94	0.64	0.78	0.93
eGFR + DS	0.92	0.95	0.97	0.71	0.84	0.97
eGFR + ICD	0.9	0.93	0.96	0.67	0.81	0.95
eGFR + ICD + DS	0.91	0.94	0.97	0.69	0.83	0.96

Supplemental 12: Detailed performance characteristics for different generalized linear model networks for identification of CKD and NKD. Including laboratory values from previous hospital stays.

CKD	Sensitivity	Specificity	PPV	NPV	F1-score	Accuracy
ICD + DS	0.94	0.89	0.86	0.95	0.9	0.91
DS	0.97	0.9	0.88	0.98	0.92	0.93
ICD	0.96	0.9	0.88	0.96	0.92	0.92
eGFR_admission	0.97	0.88	0.86	0.97	0.91	0.92
eGFR_admission + ICD	0.97	0.89	0.87	0.98	0.92	0.92
eGFR_admission + ICD + DS	0.94	0.88	0.85	0.95	0.9	0.9
eGFR_admission + DS	0.97	0.91	0.89	0.98	0.93	0.94
eGFR_discharge	0.96	0.89	0.87	0.96	0.91	0.92
eGFR_discharge + ICD	0.93	0.89	0.86	0.94	0.89	0.9
eGFR_discharge + ICD + DS	0.94	0.88	0.85	0.95	0.9	0.9
eGFR_discharge + DS	0.94	0.9	0.88	0.95	0.91	0.92
eGFR	0.94	0.88	0.85	0.95	0.9	0.9
eGFR + ICD	0.93	0.9	0.88	0.94	0.9	0.91

eGFR + ICD + DS	0.94	0.92	0.9	0.95	0.92	0.93
eGFR + DS	0.94	0.92	0.9	0.95	0.92	0.93
**NKD**						
ICD	0.97	0.98	0.94	0.99	0.95	0.98
DS	0.97	0.98	0.94	0.99	0.95	0.98
ICD + DS	0.93	0.98	0.93	0.98	0.93	0.97
eGFR_admission	0.97	0.96	0.85	0.99	0.91	0.96
eGFR_admission + DS	0.97	0.98	0.94	0.99	0.95	0.98
eGFR_admission + ICD	0.97	0.98	0.94	0.99	0.95	0.98
eGFR_admission + ICD + DS	0.93	0.98	0.93	0.98	0.93	0.97
eGFR_discharge	0.97	0.96	0.85	0.99	0.91	0.96
eGFR_discharge + DS	0.97	0.98	0.94	0.99	0.95	0.98
eGFR_discharge + ICD	0.97	0.98	0.94	0.99	0.95	0.98
eGFR_discharge + ICD + DS	0.93	0.98	0.93	0.98	0.93	0.97
eGFR	0.97	0.96	0.85	0.99	0.91	0.96
eGFR+ DS	0.97	0.98	0.94	0.99	0.95	0.98
eGFR + ICD	0.97	0.98	0.94	0.99	0.95	0.98
eGFR + ICD + DS	0.93	0.98	0.93	0.98	0.93	0.97

Supplemental 13: Detailed AUC-ROC and -PR for different generalized linear model networks for identification of CKD and NKD. Including laboratory values from previous hospital stays.

CKD	ROC lower bound	ROC	ROC upper bound	PR lower bound	PR	PR upper bound
ICD + DS	0.94	0.97	1	0.93	0.97	1
DS	0.94	0.97	1	0.92	0.97	1
ICD	0.93	0.96	0.99	0.91	0.96	1
eGFR_admission	0.93	0.96	1	0.91	0.96	1
eGFR_admission + ICD	0.93	0.97	1	0.92	0.96	1
eGFR_admission + ICD + DS	0.94	0.97	1	0.93	0.97	1
eGFR_admission + DS	0.94	0.97	1	0.92	0.97	1
eGFR_discharge	0.93	0.96	0.99	0.91	0.96	1
eGFR_discharge + ICD	0.93	0.96	0.99	0.92	0.96	1
eGFR_discharge + ICD + DS	0.94	0.97	1	0.93	0.97	1
eGFR_discharge + DS	0.94	0.97	1	0.92	0.97	1
eGFR	0.93	0.96	0.99	0.9	0.95	1
eGFR + ICD	0.93	0.96	0.99	0.92	0.96	1

eGFR + ICD + DS	0.94	0.97	0.99	0.92	0.96	1
eGFR + DS	0.94	0.97	1	0.92	0.96	1
**NKD**						
ICD	0.99	0.99	1	0.93	0.98	1
DS	0.99	1	1	0.95	0.99	1
ICD + DS	0.99	1	1	0.95	0.99	1
eGFR_admission	0.98	0.99	1	0.87	0.95	1
eGFR_admission + DS	0.99	1	1	0.95	0.99	1
eGFR_admission + ICD	0.99	0.99	1	0.93	0.98	1
eGFR_admission + ICD + DS	0.99	1	1	0.95	0.99	1
eGFR_discharge	0.98	0.99	1	0.87	0.95	1
eGFR_discharge + DS	0.99	1	1	0.95	0.99	1
eGFR_discharge + ICD	0.99	0.99	1	0.93	0.98	1
eGFR_discharge + ICD + DS	0.99	1	1	0.95	0.99	1
eGFR	0.98	0.99	1	0.89	0.96	1
eGFR + DS	0.99	1	1	0.96	0.99	1
eGFR + ICD	0.99	0.99	1	0.93	0.98	1
eGFR + ICD + DS	0.99	1	1	0.96	0.99	1

Supplemental 14: Detailed performance characteristics for different generalized linear model networks for identification of CKD and NKD. Only data from the index hospital stay included.

CKD	Sensitivity	Specificity	PPV	NPV	F1-score	Accu-racy
ICD + DS	0.94	0.88	0.85	0.95	0.9	0.9
DS	0.96	0.89	0.87	0.96	0.91	0.92
ICD	0.96	0.86	0.84	0.96	0.9	0.9
eGFR_admission	0.96	0.85	0.83	0.96	0.89	0.9
eGFR_admission + ICD	0.97	0.83	0.81	0.97	0.89	0.89
eGFR_admission + ICD + DS	0.97	0.85	0.84	0.97	0.9	0.9
eGFR_admission + DS	0.97	0.88	0.86	0.97	0.91	0.92
eGFR_discharge	0.94	0.88	0.85	0.95	0.9	0.9
eGFR_discharge + ICD	0.96	0.88	0.86	0.96	0.9	0.91
eGFR_discharge + ICD + DS	0.96	0.86	0.84	0.96	0.9	0.9
eGFR_discharge + DS	0.97	0.88	0.86	0.97	0.91	0.92
eGFR	0.9	0.88	0.85	0.92	0.87	0.88
eGFR + ICD	0.93	0.85	0.83	0.94	0.88	0.88
eGFR + ICD + DS	0.94	0.89	0.86	0.95	0.9	0.91
eGFR + DS	0.96	0.88	0.86	0.96	0.9	0.91

NKD						
ICD	0.57	0.92	0.63	0.9	0.6	0.85
DS	0.8	0.91	0.69	0.95	0.74	0.89
ICD + DS	0.77	0.91	0.68	0.94	0.72	0.88
eGFR_admission	0.37	0.95	0.65	0.86	0.47	0.84
eGFR_admission + DS	0.87	0.92	0.72	0.97	0.79	0.91
eGFR_admission + ICD	0.57	0.92	0.63	0.9	0.6	0.85
eGFR_admission + ICD + DS	0.83	0.92	0.71	0.96	0.77	0.9
eGFR_discharge	0.53	0.93	0.64	0.89	0.58	0.85
eGFR_discharge + DS	0.9	0.91	0.71	0.97	0.79	0.91
eGFR_discharge + ICD	0.57	0.92	0.63	0.9	0.6	0.85
eGFR_discharge + ICD + DS	0.87	0.91	0.7	0.97	0.78	0.9
eGFR	0.5	0.94	0.65	0.89	0.57	0.85
eGFR+ DS	0.97	0.9	0.69	0.99	0.81	0.91
eGFR + ICD	0.53	0.93	0.64	0.89	0.58	0.85
eGFR + ICD + DS	0.97	0.9	0.69	0.99	0.81	0.91

Supplemental 15: Detailed AUC-ROC and -PR for different generalized linear model networks for identification of CKD and NKD. Only data from the index hospital stay included.

**CKD**	**ROC lower bound**	**ROC**	**ROC upper bound**	**PR lower bound**	**PR**	**PR upper bound**
ICD + DS	0.95	0.97	1	0.93	0.97	1
DS	0.94	0.97	1	0.92	0.96	1
ICD	0.93	0.96	0.99	0.91	0.96	1
eGFR_admission	0.92	0.95	0.99	0.9	0.95	1
eGFR_admission + ICD	0.93	0.96	1	0.92	0.97	1
eGFR_admission + ICD + DS	0.95	0.97	1	0.93	0.97	1
eGFR_admission + DS	0.94	0.97	1	0.92	0.96	1
eGFR_discharge	0.92	0.96	0.99	0.9	0.95	1
eGFR_discharge + ICD	0.93	0.96	0.99	0.91	0.96	1
eGFR_discharge + ICD + DS	0.95	0.97	1	0.93	0.97	1
eGFR_discharge + DS	0.94	0.97	0.99	0.91	0.96	1
eGFR	0.91	0.95	0.98	0.89	0.94	1
eGFR + ICD	0.92	0.96	0.99	0.91	0.95	1
eGFR + ICD + DS	0.94	0.97	0.99	0.92	0.97	1
eGFR + DS	0.94	0.97	0.99	0.92	0.96	1
**NKD**						

ICD	0.9	0.93	0.97	0.46	0.64	0.81
DS	0.92	0.95	0.98	0.54	0.7	0.87
ICD + DS	0.92	0.95	0.98	0.53	0.69	0.86
eGFR_admission	0.88	0.92	0.96	0.52	0.68	0.85
eGFR_admission + DS	0.92	0.95	0.98	0.58	0.74	0.9
eGFR_admission + ICD	0.9	0.94	0.97	0.47	0.64	0.81
eGFR_admission + ICD + DS	0.92	0.95	0.98	0.57	0.73	0.89
eGFR_discharge	0.89	0.93	0.97	0.44	0.62	0.79
eGFR_discharge + DS	0.92	0.95	0.98	0.58	0.73	0.89
eGFR_discharge + ICD	0.9	0.93	0.97	0.45	0.63	0.8
eGFR_discharge + ICD + DS	0.92	0.95	0.98	0.56	0.72	0.88
eGFR	0.89	0.93	0.97	0.53	0.69	0.86
eGFR + DS	0.92	0.95	0.98	0.63	0.78	0.93
eGFR + ICD	0.91	0.94	0.98	0.56	0.72	0.88
eGFR + ICD + DS	0.92	0.95	0.98	0.63	0.78	0.93

Supplemental 16: Detailed performance characteristics for different random forest models for identification of CKD and NKD. Including laboratory values from previous hospital stays.

CKD	Sensitivity	Specificity	PPV	NPV	F1-score	Accuracy
ICD + DS	0.94	0.86	0.84	0.95	0.89	0.9

DS	0.94	0.88	0.85	0.95	0.9	0.9
ICD	0.94	0.89	0.86	0.95	0.9	0.91
eGFR_admission	0.96	0.89	0.87	0.96	0.91	0.92
eGFR_admission + ICD	0.94	0.86	0.84	0.95	0.89	0.9
eGFR_admission + ICD + DS	0.94	0.88	0.85	0.95	0.9	0.9
eGFR_admission + DS	0.94	0.88	0.85	0.95	0.9	0.9
eGFR_discharge	0.96	0.88	0.86	0.96	0.9	0.91
eGFR_discharge + ICD	0.94	0.88	0.85	0.95	0.9	0.9
eGFR_discharge + ICD + DS	0.94	0.88	0.85	0.95	0.9	0.9
eGFR_discharge + DS	0.94	0.86	0.84	0.95	0.89	0.9
eGFR	0.94	0.89	0.86	0.95	0.9	0.91
eGFR + ICD	0.94	0.86	0.84	0.95	0.89	0.9
eGFR + ICD + DS	0.94	0.89	0.86	0.95	0.9	0.91
eGFR + DS	0.94	0.88	0.85	0.95	0.9	0.9
**NKD**						
ICD	0.9	0.98	0.9	0.98	0.9	0.96
DS	0.9	0.98	0.93	0.98	0.92	0.97

ICD + DS	0.9	0.98	0.93	0.98	0.92	0.97
eGFR_admission	0.87	0.97	0.87	0.97	0.87	0.95
eGFR_admission + DS	0.9	0.98	0.93	0.98	0.92	0.97
eGFR_admission + ICD	0.9	0.98	0.9	0.98	0.9	0.96
eGFR_admission + ICD + DS	0.9	0.98	0.93	0.98	0.92	0.97
eGFR_discharge	0.87	0.97	0.87	0.97	0.87	0.95
eGFR_discharge + DS	0.9	0.98	0.93	0.98	0.92	0.97
eGFR_discharge + ICD	0.9	0.98	0.9	0.98	0.9	0.96
eGFR_discharge + ICD + DS	0.9	0.98	0.93	0.98	0.92	0.97
eGFR	0.9	0.96	0.84	0.98	0.87	0.95
eGFR+ DS	0.9	0.99	0.96	0.98	0.93	0.97
eGFR + ICD	0.9	0.98	0.93	0.98	0.92	0.97
eGFR + ICD + DS	0.9	0.99	0.96	0.98	0.93	0.97

Supplemental 17: Detailed AUC-ROC and -PR for different random forest models for identification of CKD and NKD. Including laboratory values from previous hospital stays.

**CKD**	**ROC lower bound**	**ROC**	**ROC upper bound**	**PR lower bound**	**PR**	**PR upper bound**

ICD + DS	0.94	0.97	1	0.92	0.96	1
DS	0.93	0.96	0.99	0.9	0.95	1
ICD	0.93	0.96	0.99	0.91	0.96	1
eGFR_admission	0.93	0.96	0.99	0.9	0.95	1
eGFR_admission + ICD	0.93	0.96	0.99	0.91	0.96	1
eGFR_admission + ICD + DS	0.94	0.97	0.99	0.91	0.96	1
eGFR_admission + DS	0.93	0.96	1	0.91	0.96	1
eGFR_discharge	0.94	0.97	0.99	0.91	0.96	1
eGFR_discharge + ICD	0.94	0.97	0.99	0.92	0.96	1
eGFR_discharge + ICD + DS	0.94	0.97	0.99	0.92	0.96	1
eGFR_discharge + DS	0.93	0.96	0.99	0.89	0.95	1
eGFR	0.93	0.96	0.99	0.89	0.94	1
eGFR + ICD	0.93	0.96	0.99	0.9	0.95	1
eGFR + ICD + DS	0.93	0.97	1	0.91	0.96	1
eGFR + DS	0.94	0.97	1	0.91	0.96	1
**NKD**						
ICD	0.97	0.99	1	0.88	0.96	1
DS	0.98	0.99	1	0.9	0.96	1
ICD + DS	0.98	0.99	1	0.9	0.96	1

eGFR_admission	0.98	0.99	1	0.88	0.95	1
eGFR_admission + DS	0.97	0.99	1	0.89	0.96	1
eGFR_admission + ICD	0.97	0.99	1	0.89	0.96	1
eGFR_admission + ICD + DS	0.97	0.99	1	0.89	0.96	1
eGFR_discharge	0.97	0.99	1	0.87	0.95	1
eGFR_discharge + DS	0.97	0.99	1	0.89	0.96	1
eGFR_discharge + ICD	0.97	0.99	1	0.88	0.96	1
eGFR_discharge + ICD + DS	0.98	0.99	1	0.9	0.97	1
eGFR	0.97	0.99	1	0.86	0.95	1
eGFR + DS	0.98	0.99	1	0.92	0.98	1
eGFR + ICD	0.97	0.99	1	0.89	0.96	1
eGFR + ICD + DS	0.98	0.99	1	0.92	0.98	1

Supplemental 18: Detailed performance characteristics for different random forest models for identification of CKD and NKD. Only data from the index hospital stay included.

CKD	Sensitivity	Specificity	PPV	NPV	F1-score	Accuracy
ICD + DS	0.96	0.85	0.83	0.96	0.89	0.9
DS	0.96	0.86	0.84	0.96	0.9	0.9
ICD	0.96	0.88	0.86	0.96	0.9	0.91
eGFR_admission	0.91	0.85	0.83	0.93	0.87	0.88

eGFR_admission + ICD	0.96	0.86	0.84	0.96	0.9	0.9
eGFR_admission + ICD + DS	0.97	0.89	0.87	0.98	0.92	0.92
eGFR_admission + DS	0.96	0.89	0.87	0.96	0.91	0.92
eGFR_discharge	0.91	0.84	0.82	0.92	0.86	0.87
eGFR_discharge + ICD	0.96	0.86	0.84	0.96	0.9	0.9
eGFR_discharge + ICD + DS	0.97	0.86	0.85	0.97	0.9	0.91
eGFR_discharge + DS	0.96	0.89	0.87	0.96	0.91	0.92
eGFR	0.94	0.89	0.86	0.95	0.9	0.91
eGFR + ICD	0.93	0.88	0.85	0.94	0.89	0.9
eGFR + ICD + DS	0.94	0.89	0.86	0.95	0.9	0.91
eGFR + DS	0.96	0.89	0.87	0.96	0.91	0.92
**NKD**						
ICD	0.53	0.9	0.57	0.89	0.55	0.83
DS	0.77	0.9	0.66	0.94	0.71	0.88
ICD + DS	0.77	0.9	0.66	0.94	0.71	0.88
eGFR_admission	0.57	0.89	0.55	0.9	0.56	0.83
eGFR_admission + DS	0.8	0.9	0.67	0.95	0.73	0.88
eGFR_admission + ICD	0.6	0.91	0.62	0.91	0.61	0.85

eGFR_admission + ICD + DS	0.8	0.9	0.67	0.95	0.73	0.88
eGFR_discharge	0.43	0.9	0.5	0.87	0.46	0.81
eGFR_discharge + DS	0.8	0.9	0.67	0.95	0.73	0.88
eGFR_discharge + ICD	0.57	0.92	0.63	0.9	0.6	0.85
eGFR_discharge + ICD + DS	0.8	0.9	0.67	0.95	0.73	0.88
eGFR	0.43	0.9	0.5	0.87	0.46	0.81
eGFR+ DS	0.8	0.91	0.69	0.95	0.74	0.89
eGFR + ICD	0.57	0.92	0.63	0.9	0.6	0.85
eGFR + ICD + DS	0.8	0.91	0.69	0.95	0.74	0.89

Supplemental 19: Detailed AUC-ROC and -PR for different random forest models for identification of CKD and NKD. Only data from the index hospital stay included.

**CKD**	**ROC lower bound**	**ROC**	**ROC upper bound**	**PR lower bound**	**PR**	**PR upper bound**
ICD + DS	0.95	0.97	1	0.93	0.97	1
DS	0.95	0.97	1	0.87	0.93	0.99
ICD	0.93	0.96	0.99	0.91	0.96	1
eGFR_admission	0.92	0.96	0.99	0.9	0.95	1

eGFR_admission + ICD	0.93	0.96	0.99	0.92	0.96	1
eGFR_admission + ICD + DS	0.95	0.97	1	0.93	0.97	1
eGFR_admission + DS	0.95	0.97	1	0.92	0.96	1
eGFR_discharge	0.92	0.96	0.99	0.91	0.95	1
eGFR_discharge + ICD	0.93	0.96	0.99	0.91	0.96	1
eGFR_discharge + ICD + DS	0.95	0.97	1	0.93	0.97	1
eGFR_discharge + DS	0.94	0.97	1	0.85	0.92	0.98
eGFR	0.92	0.95	0.99	0.85	0.92	0.98
eGFR + ICD	0.93	0.96	0.99	0.9	0.95	1
eGFR + ICD + DS	0.95	0.97	0.99	0.92	0.96	1
eGFR + DS	0.95	0.97	0.99	0.84	0.91	0.98
**NKD**						
ICD	0.9	0.93	0.97	0.35	0.53	0.71
DS	0.92	0.95	0.98	0.45	0.62	0.79
ICD + DS	0.91	0.95	0.98	0.41	0.59	0.76
eGFR_admission	0.88	0.92	0.96	0.36	0.54	0.71
eGFR_admission + DS	0.92	0.95	0.98	0.45	0.62	0.8
eGFR_admission + ICD	0.9	0.93	0.97	0.38	0.56	0.73
eGFR_admission + ICD + DS	0.91	0.95	0.98	0.39	0.57	0.75

eGFR_discharge	0.88	0.92	0.96	0.36	0.54	0.72
eGFR_discharge + DS	0.92	0.95	0.98	0.43	0.61	0.78
eGFR_discharge + ICD	0.9	0.93	0.97	0.39	0.57	0.74
eGFR_discharge + ICD + DS	0.91	0.95	0.98	0.41	0.58	0.76
eGFR	0.89	0.93	0.97	0.38	0.56	0.73
eGFR + DS	0.93	0.96	0.99	0.46	0.63	0.81
eGFR + ICD	0.91	0.94	0.98	0.4	0.57	0.75
eGFR + ICD + DS	0.93	0.96	0.99	0.42	0.6	0.77

Supplemental 20: Detailed performance characteristics for different neural networks models for identification of CKD and NKD. Including laboratory values from previous hospital stays.

CKD	Sensitivity	Specificity	PPV	NPV	F1-score	Accu-racy
ICD + DS	0.93	0.81	0.79	0.93	0.85	0.86
DS	0.96	0.9	0.88	0.96	0.92	0.92
ICD	0.9	0.92	0.9	0.92	0.9	0.91
eGFR_admission	0.93	0.81	0.79	0.93	0.85	0.86
eGFR_admission + ICD	0.91	0.92	0.9	0.93	0.91	0.92
eGFR_admission + ICD + DS	0.91	0.93	0.91	0.93	0.91	0.92
eGFR_admission + DS	0.94	0.85	0.83	0.95	0.88	0.89
eGFR_discharge	0.94	0.89	0.86	0.95	0.9	0.91
eGFR_discharge + ICD	0.79	0.94	0.92	0.86	0.85	0.88
eGFR_discharge + ICD + DS	0.93	0.89	0.86	0.94	0.89	0.9
eGFR_discharge + DS	0.94	0.89	0.86	0.95	0.9	0.91
eGFR	0.94	0.9	0.88	0.95	0.91	0.92
eGFR + ICD	0.91	0.94	0.93	0.93	0.92	0.93

eGFR + ICD + DS	0.93	0.9	0.88	0.94	0.9	0.91
eGFR + DS	0.96	0.82	0.8	0.96	0.87	0.88
**NKD**						
ICD	0.93	0.98	0.93	0.98	0.93	0.97
DS	0.97	0.98	0.91	0.99	0.94	0.97
ICD + DS	1	0.98	0.94	1	0.97	0.99
eGFR_admission	0.97	0.95	0.83	0.99	0.89	0.96
eGFR_admission + DS	0.9	0.99	0.96	0.98	0.93	0.97
eGFR_admission + ICD	0.97	0.98	0.94	0.99	0.95	0.98
eGFR_admission + ICD + DS	0.9	0.98	0.93	0.98	0.92	0.97
eGFR_discharge	0.97	0.95	0.83	0.99	0.89	0.96
eGFR_discharge + DS	0.97	0.98	0.91	0.99	0.94	0.97
eGFR_discharge + ICD	0.93	0.98	0.9	0.98	0.92	0.97
eGFR_discharge + ICD + DS	0.87	0.94	0.79	0.97	0.83	0.93
eGFR	0.97	0.97	0.88	0.99	0.92	0.97
eGFR+ DS	0.77	0.97	0.85	0.95	0.81	0.93
eGFR + ICD	0.93	0.98	0.93	0.98	0.93	0.97
eGFR + ICD + DS	0.97	0.98	0.91	0.99	0.94	0.97

Supplemental 21: Detailed AUC-ROC and -PR for for different neural networks models for identification of CKD and NKD. Including laboratory values from previous hospital stays.

CKD	ROC lower bound	ROC	ROC upper bound	PR lower bound	PR	PR upper bound
ICD + DS	0.82	0.87	0.93	0.78	0.86	0.95
DS	0.94	0.96	0.99	0.91	0.96	1
ICD	0.9	0.94	0.98	0.89	0.94	1
eGFR_admission	0.81	0.87	0.92	0.79	0.87	0.95
eGFR_admission + ICD	0.92	0.96	0.99	0.89	0.95	1
eGFR_admission + ICD + DS	0.93	0.96	0.99	0.9	0.95	1
eGFR_admission + DS	0.93	0.96	0.99	0.9	0.95	1
eGFR_discharge	0.93	0.96	0.99	0.91	0.96	1
eGFR_discharge + ICD	0.91	0.95	0.98	0.89	0.94	1
eGFR_discharge + ICD + DS	0.93	0.96	0.99	0.9	0.95	1
eGFR_discharge + DS	0.92	0.95	0.99	0.89	0.95	1
eGFR	0.91	0.94	0.98	0.89	0.94	1
eGFR + ICD	0.93	0.96	0.99	0.91	0.96	1

eGFR + ICD + DS	0.92	0.95	0.99	0.89	0.95	1
eGFR + DS	0.93	0.96	0.99	0.9	0.95	1
**NKD**						
ICD	0.92	0.97	1	0.89	0.96	1
DS	0.99	1	1	0.96	0.99	1
ICD + DS	1	1	1	0.98	1	1
eGFR_admission	0.91	0.95	0.99	0.64	0.78	0.93
eGFR_admission + DS	0.94	0.98	1	0.9	0.97	1
eGFR_admission + ICD	0.98	0.99	1	0.9	0.97	1
eGFR_admission + ICD + DS	0.99	0.99	1	0.92	0.97	1
eGFR_discharge	0.95	0.97	1	0.64	0.78	0.93
eGFR_discharge + DS	1	1	1	0.97	1	1
eGFR_discharge + ICD	0.96	0.98	1	0.84	0.93	1
eGFR_discharge + ICD + DS	0.91	0.95	0.98	0.59	0.75	0.9
eGFR	0.97	0.99	1	0.84	0.93	1
eGFR + DS	0.95	0.97	0.99	0.72	0.85	0.97
eGFR + ICD	0.94	0.98	1	0.86	0.94	1
eGFR + ICD + DS	0.97	0.99	1	0.8	0.9	1

Supplemental 22: Detailed performance characteristics for different neural networks models for identification of CKD and NKD. Only data from the index hospital stay included.

CKD	Sensitivity	Specificity	PPV	NPV	F1-score	Accu-racy
ICD + DS	0.75	0.97	0.94	0.83	0.84	0.87
DS	0.82	0.94	0.92	0.87	0.87	0.89
ICD	0.9	0.93	0.91	0.92	0.9	0.92
eGFR_admission	0.82	0.92	0.89	0.87	0.85	0.88
eGFR_admission + ICD	0.81	0.95	0.93	0.87	0.87	0.89
eGFR_admission + ICD + DS	0.85	0.81	0.77	0.88	0.81	0.83
eGFR_admission + DS	0.96	0.88	0.86	0.96	0.9	0.91
eGFR_discharge	0.94	0.83	0.81	0.95	0.87	0.88
eGFR_discharge + ICD	0.97	0.78	0.78	0.97	0.86	0.87
eGFR_discharge + ICD + DS	0.97	0.85	0.84	0.97	0.9	0.9
eGFR_discharge + DS	0.97	0.78	0.78	0.97	0.86	0.87
eGFR	0.97	0.84	0.82	0.97	0.89	0.9
eGFR + ICD	0.94	0.86	0.84	0.95	0.89	0.9
eGFR + ICD + DS	0.9	0.86	0.84	0.92	0.87	0.88
eGFR + DS	0.87	0.89	0.86	0.9	0.86	0.88

**NKD**						
ICD	0.63	0.9	0.59	0.91	0.61	0.85
DS	0.97	0.89	0.67	0.99	0.79	0.9
ICD + DS	0.87	0.9	0.68	0.97	0.76	0.9
eGFR_admission	0.93	0.83	0.56	0.98	0.7	0.85
eGFR_admission + DS	0.9	0.88	0.64	0.97	0.75	0.88
eGFR_admission + ICD	0.97	0.83	0.57	0.99	0.72	0.85
eGFR_admission + ICD + DS	0	1	NA	0.81	NA	0.81
eGFR_discharge	0.9	0.84	0.57	0.97	0.7	0.85
eGFR_discharge + DS	0.83	0.89	0.64	0.96	0.72	0.88
eGFR_discharge + ICD	0	1	NA	0.81	NA	0.81
eGFR_discharge + ICD + DS	0.97	0.89	0.67	0.99	0.79	0.9
eGFR	0.83	0.84	0.56	0.95	0.67	0.84
eGFR+ DS	0.77	0.9	0.66	0.94	0.71	0.88
eGFR + ICD	0	1	NA	0.81	NA	0.81
eGFR + ICD + DS	0.53	0.9	0.55	0.89	0.54	0.83

Supplemental 23: Detailed AUC-ROC and -PR for for different neural networks models for identification of CKD and NKD. Only data from the index hospital stay included.

CKD	ROC lower bound	ROC	ROC upper bound	PR lower bound	PR	PR upper bound
ICD + DS	0.88	0.93	0.97	0.87	0.93	0.99
DS	0.88	0.93	0.97	0.87	0.93	0.99
ICD	0.9	0.94	0.98	0.87	0.93	0.99
eGFR_admission	0.9	0.94	0.98	0.86	0.92	0.99
eGFR_admission + ICD	0.92	0.95	0.99	0.89	0.94	1
eGFR_admission + ICD + DS	0.85	0.9	0.95	0.84	0.91	0.98
eGFR_admission + DS	0.93	0.96	0.99	0.91	0.96	1
eGFR_discharge	0.9	0.94	0.98	0.86	0.92	0.99
eGFR_discharge + ICD	0.9	0.94	0.97	0.87	0.93	0.99
eGFR_discharge + ICD + DS	0.95	0.97	0.99	0.92	0.97	1
eGFR_discharge + DS	0.9	0.94	0.98	0.86	0.93	0.99
eGFR	0.85	0.9	0.95	0.81	0.89	0.96
eGFR + ICD	0.92	0.96	0.99	0.91	0.96	1
eGFR + ICD + DS	0.88	0.92	0.97	0.87	0.93	0.99
eGFR + DS	0.91	0.95	0.98	0.88	0.94	1
**NKD**						

ICD	0.89	0.93	0.97	0.47	0.64	0.81
DS	0.88	0.93	0.97	0.64	0.78	0.93
ICD + DS	0.89	0.93	0.97	0.49	0.66	0.83
eGFR_admission	0.82	0.88	0.94	0.6	0.75	0.91
eGFR_admission + DS	0.9	0.94	0.99	0.62	0.77	0.92
eGFR_admission + ICD	0.85	0.9	0.94	0.62	0.77	0.92
eGFR_admission + ICD + DS	0.75	0.81	0.88	0.51	0.68	0.85
eGFR_discharge	0.89	0.93	0.97	0.49	0.66	0.83
eGFR_discharge + DS	0.9	0.94	0.97	0.56	0.72	0.88
eGFR_discharge + ICD	0.85	0.89	0.94	0.57	0.73	0.89
eGFR_discharge + ICD + DS	0.92	0.95	0.98	0.66	0.8	0.95
eGFR	0.86	0.91	0.95	0.38	0.56	0.74
eGFR + DS	0.88	0.93	0.97	0.46	0.63	0.8
eGFR + ICD	0.5	0.5	0.5	0.42	0.6	0.77
eGFR + ICD + DS	0.65	0.74	0.83	0.42	0.6	0.77

Supplemental 24: Detailed performance characteristics for different generalized linear models for identification of CKD and NKD. Including laboratory values from previous hospital stays.

**CKD**	**Sensitivity**	**Specificity**	**PPV**	**NPV**	**F1-score**	**Accuracy**
ICD + DS	0.94	0.88	0.85	0.95	0.9	0.9

DS	0.94	0.9	0.88	0.95	0.91	0.92
ICD	0.93	0.88	0.85	0.94	0.89	0.9
eGFR_admission	0.94	0.88	0.85	0.95	0.9	0.9
eGFR_admission + ICD	0.93	0.88	0.85	0.94	0.89	0.9
eGFR_admission + ICD + DS	0.94	0.88	0.85	0.95	0.9	0.9
eGFR_admission + DS	0.96	0.9	0.88	0.96	0.92	0.92
eGFR_discharge	0.94	0.88	0.85	0.95	0.9	0.9
eGFR_discharge + ICD	0.93	0.86	0.84	0.94	0.88	0.89
eGFR_discharge + ICD + DS	0.94	0.89	0.86	0.95	0.9	0.91
eGFR_discharge + DS	0.94	0.9	0.88	0.95	0.91	0.92
eGFR	0.94	0.89	0.86	0.95	0.9	0.91
eGFR + ICD	0.93	0.9	0.88	0.94	0.9	0.91
eGFR + ICD + DS	0.93	0.89	0.86	0.94	0.89	0.9
eGFR + DS	0.94	0.9	0.88	0.95	0.91	0.92
**NKD**						
ICD	0.93	0.98	0.93	0.98	0.93	0.97
DS	0.3	1	1	0.86	0.46	0.87

ICD + DS	0.33	0.99	0.91	0.86	0.49	0.87
eGFR_admission	0.93	0.96	0.85	0.98	0.89	0.96
eGFR_admission + DS	0.9	0.98	0.9	0.98	0.9	0.96
eGFR_admission + ICD	0.93	0.98	0.93	0.98	0.93	0.97
eGFR_admission + ICD + DS	0.9	0.98	0.93	0.98	0.92	0.97
eGFR_discharge	0.93	0.96	0.85	0.98	0.89	0.96
eGFR_discharge + DS	0.73	0.96	0.81	0.94	0.77	0.92
eGFR_discharge + ICD	0.93	0.98	0.93	0.98	0.93	0.97
eGFR_discharge + ICD + DS	0.73	0.98	0.92	0.94	0.81	0.94
eGFR	0.93	0.95	0.82	0.98	0.87	0.95
eGFR+ DS	0.9	0.98	0.9	0.98	0.9	0.96
eGFR + ICD	0.93	0.96	0.85	0.98	0.89	0.96
eGFR + ICD + DS	0.9	0.98	0.93	0.98	0.92	0.97

Supplemental 25: Detailed AUC-ROC and -PR for for different generalized linear models for identification of CKD and NKD. Including laboratory values from previous hospital stays.

**CKD**	**ROC lower bound**	**ROC**	**ROC upper bound**	**PR lower bound**	**PR**	**PR upper bound**

ICD + DS	0.94	0.97	0.99	0.92	0.97	1
DS	0.94	0.97	0.99	0.92	0.96	1
ICD	0.93	0.96	0.99	0.92	0.96	1
eGFR_admission	0.93	0.96	0.99	0.91	0.96	1
eGFR_admission + ICD	0.93	0.96	0.99	0.92	0.96	1
eGFR_admission + ICD + DS	0.94	0.97	1	0.92	0.97	1
eGFR_admission + DS	0.94	0.97	0.99	0.92	0.96	1
eGFR_discharge	0.93	0.96	0.99	0.91	0.96	1
eGFR_discharge + ICD	0.93	0.96	0.99	0.92	0.96	1
eGFR_discharge + ICD + DS	0.94	0.97	0.99	0.92	0.97	1
eGFR_discharge + DS	0.94	0.97	0.99	0.92	0.96	1
eGFR	0.93	0.96	0.99	0.91	0.96	1
eGFR + ICD	0.93	0.96	0.99	0.92	0.96	1
eGFR + ICD + DS	0.94	0.97	0.99	0.92	0.96	1
eGFR + DS	0.94	0.97	0.99	0.92	0.96	1
**NKD**						
ICD	0.93	0.97	1	0.73	0.85	0.98
DS	0.57	0.65	0.73	0.56	0.72	0.88
ICD + DS	0.58	0.66	0.75	0.52	0.69	0.85

eGFR_admission	0.96	0.98	1	0.68	0.82	0.96
eGFR_admission + DS	0.96	0.98	1	0.7	0.83	0.96
eGFR_admission + ICD	0.93	0.97	1	0.73	0.85	0.98
eGFR_admission + ICD + DS	0.93	0.97	1	0.71	0.84	0.97
eGFR_discharge	0.96	0.98	1	0.68	0.82	0.96
eGFR_discharge + DS	0.76	0.85	0.93	0.66	0.8	0.94
eGFR_discharge + ICD	0.93	0.97	1	0.73	0.85	0.98
eGFR_discharge + ICD + DS	0.78	0.86	0.94	0.72	0.85	0.98
eGFR	0.94	0.97	1	0.61	0.76	0.91
eGFR + DS	0.95	0.98	1	0.65	0.8	0.94
eGFR + ICD	0.91	0.95	1	0.57	0.73	0.89
eGFR + ICD + DS	0.92	0.96	1	0.65	0.79	0.94

Supplemental 26: Detailed performance characteristics for different generalized linear models for identification of CKD and NKD. Only data from the index hospital stay included.

**CKD**	**Sensitivity**	**Specificity**	**PPV**	**NPV**	**F1-score**	**Accuracy**
ICD + DS	0.97	0.86	0.85	0.97	0.9	0.91
DS	0.97	0.86	0.85	0.97	0.9	0.91
ICD	0.96	0.88	0.86	0.96	0.9	0.91
eGFR_admission	0.97	0.86	0.85	0.97	0.9	0.91

eGFR_admission + ICD	0.97	0.86	0.85	0.97	0.9	0.91
eGFR_admission + ICD + DS	0.97	0.85	0.84	0.97	0.9	0.9
eGFR_admission + DS	0.97	0.88	0.86	0.97	0.91	0.92
eGFR_discharge	0.93	0.88	0.85	0.94	0.89	0.9
eGFR_discharge + ICD	0.96	0.88	0.86	0.96	0.9	0.91
eGFR_discharge + ICD + DS	0.97	0.86	0.85	0.97	0.9	0.91
eGFR_discharge + DS	0.97	0.86	0.85	0.97	0.9	0.91
eGFR	0.94	0.85	0.83	0.95	0.88	0.89
eGFR + ICD	0.96	0.86	0.84	0.96	0.9	0.9
eGFR + ICD + DS	0.96	0.85	0.83	0.96	0.89	0.9
eGFR + DS	0.96	0.88	0.86	0.96	0.9	0.91
**NKD**						
ICD	0.57	0.92	0.63	0.9	0.6	0.85
DS	0.9	0.9	0.69	0.97	0.78	0.9
ICD + DS	0.87	0.9	0.68	0.97	0.76	0.9
eGFR_admission	0.57	0.91	0.61	0.9	0.59	0.85
eGFR_admission + DS	0.9	0.9	0.69	0.97	0.78	0.9
eGFR_admission + ICD	0.57	0.92	0.63	0.9	0.6	0.85

eGFR_admission + ICD + DS	0.87	0.9	0.68	0.97	0.76	0.9
eGFR_discharge	0.57	0.91	0.61	0.9	0.59	0.85
eGFR_discharge + DS	0.9	0.9	0.69	0.97	0.78	0.9
eGFR_discharge + ICD	0.57	0.92	0.63	0.9	0.6	0.85
eGFR_discharge + ICD + DS	0.87	0.9	0.68	0.97	0.76	0.9
eGFR	0.57	0.92	0.63	0.9	0.6	0.85
eGFR+ DS	0.9	0.91	0.71	0.97	0.79	0.91
eGFR + ICD	0.57	0.93	0.65	0.9	0.61	0.86
eGFR + ICD + DS	0.87	0.91	0.7	0.97	0.78	0.9

Supplemental 27: Detailed AUC-ROC and -PR for for different generalized linear models for identification of CKD and NKD. Only data from the index hospital stay included.

**CKD**	**ROC lower bound**	**ROC**	**ROC upper bound**	**PR lower bound**	**PR**	**PR upper bound**
ICD + DS	0.95	0.97	1	0.92	0.97	1
DS	0.95	0.97	1	0.92	0.96	1
ICD	0.93	0.96	0.99	0.91	0.96	1
eGFR_admission	0.92	0.96	0.99	0.9	0.95	1

eGFR_admission + ICD	0.93	0.96	0.99	0.91	0.96	1
eGFR_admission + ICD + DS	0.95	0.97	1	0.93	0.97	1
eGFR_admission + DS	0.95	0.97	1	0.92	0.96	1
eGFR_discharge	0.92	0.96	0.99	0.9	0.95	1
eGFR_discharge + ICD	0.93	0.96	0.99	0.91	0.96	1
eGFR_discharge + ICD + DS	0.95	0.97	1	0.92	0.97	1
eGFR_discharge + DS	0.94	0.97	1	0.92	0.96	1
eGFR	0.92	0.95	0.99	0.89	0.95	1
eGFR + ICD	0.93	0.96	0.99	0.91	0.96	1
eGFR + ICD + DS	0.95	0.97	0.99	0.92	0.97	1
eGFR + DS	0.95	0.97	0.99	0.92	0.96	1
**NKD**						
ICD	0.9	0.93	0.97	0.46	0.63	0.81
DS	0.92	0.95	0.98	0.54	0.7	0.87
ICD + DS	0.91	0.95	0.98	0.52	0.69	0.85
eGFR_admission	0.88	0.92	0.96	0.41	0.59	0.77
eGFR_admission + DS	0.92	0.95	0.98	0.54	0.7	0.87
eGFR_admission + ICD	0.9	0.93	0.97	0.46	0.63	0.81
eGFR_admission + ICD + DS	0.91	0.95	0.98	0.52	0.69	0.85

eGFR_discharge	0.88	0.92	0.96	0.41	0.59	0.77
eGFR_discharge + DS	0.92	0.95	0.98	0.54	0.7	0.87
eGFR_discharge + ICD	0.9	0.93	0.97	0.46	0.63	0.81
eGFR_discharge + ICD + DS	0.91	0.95	0.98	0.52	0.69	0.85
eGFR	0.89	0.93	0.97	0.46	0.63	0.8
eGFR + DS	0.93	0.96	0.99	0.67	0.81	0.95
eGFR + ICD	0.91	0.94	0.98	0.57	0.73	0.89
eGFR + ICD + DS	0.93	0.96	0.99	0.65	0.79	0.94

Supplemental 28: Detailed hyperparameters of different machine learning models. Including laboratory values from previous hospital stays.

Model	GLMNet		Random forest	Neural net-works
	alpha	lambda*	mtry	size
**CKD**				
ICD + DS	1	0	10	7
DS	1	0.08	8	9
ICD	1	0.08	4	10
eGFR_admission	1	0.01	5	10
eGFR_admission + ICD	1	0.01	6	6
eGFR_admission + ICD + DS	0.4	0	5	10
eGFR_admission + DS	1	0.01	3	7
eGFR_discharge	0.8	0	5	10
eGFR_discharge + ICD	1	0	6	6
eGFR_discharge + ICD + DS	0.9	0	3	10
eGFR_discharge + DS	0.9	0	2	5
eGFR	0.9	0.01	2	9
eGFR + ICD	0.4	0	1	6
eGFR + ICD + DS	1	0.01	5	10
eGFR + DS	1	0.01	4	10

NKD				
ICD	1	0	16	6
DS	1	0	11	9
ICD + DS	1	0	11	7
eGFR_admission	1	0	3	10
eGFR_admission + DS	1	0	12	10
eGFR_admission + ICD	1	0	14	10
eGFR_admission + ICD + DS	1	0	13	10
eGFR_discharge	1	0	2	8
eGFR_discharge + DS	1	0	12	10
eGFR_discharge + ICD	1	0	16	10
eGFR_discharge + ICD + DS	1	0	11	8
eGFR	1	0	12	9
eGFR + DS	1	0	10	6
eGFR + ICD	1	0	18	10
eGFR + ICD + DS	1	0	12	8

Supplemental 29: Detailed hyperparameters of different machine learning models. Only data from the index hospital stay included.

Model	GLMNet		

	alp ha	lambda*	Random forest<br><br>mtry	Neural net-works<br><br>size
**CKD**				
ICD + DS	0.2	0.08	2	9
DS	0.2	0.08	1	9
ICD	0.2	0.08	1	10
eGFR_admission	1	0.01	9	10
eGFR_admission + ICD	0.1	0.08	2	9
eGFR_admission + ICD + DS	0.1	0	1	8
eGFR_admission + DS	0.3	0.08	2	7
eGFR_discharge	0.1	0	10	8
eGFR_discharge + ICD	0.4	0	1	10
eGFR_discharge + ICD + DS	0.9	0	1	7
eGFR_discharge + DS	0.9	0.01	1	10
eGFR	0.3	0.08	1	8
eGFR + ICD	0.1	0.08	1	8
eGFR + ICD + DS	0.2	0.08	1	8
eGFR + DS	0.1	0	1	10
**NKD**				
ICD	0.7	0	4	3

DS	0.9	0	11	8
ICD + DS	0.9	0	10	7
eGFR_admission	0	0.04	2	2
eGFR_admission + DS	0.7	0	9	8
eGFR_admission + ICD	0.8	0	2	5
eGFR_admission + ICD + DS	0.8	0	9	10
eGFR_discharge	0.2	0	2	3
eGFR_discharge + DS	1	0	10	8
eGFR_discharge + ICD	0.7	0	2	6
eGFR_discharge + ICD + DS	1	0	5	9
eGFR	0.5	0	2	9
eGFR + DS	1	0	4	8
eGFR + ICD	0.1	0	2	1
eGFR + ICD + DS	1	0	7	10

*lambda values are rounded to the second decimal place
